# Poly(pentacenetetrone) as a High‐capacity Cathode for Sodium Batteries

**DOI:** 10.1002/advs.202500484

**Published:** 2025-03-26

**Authors:** Chinmaya Mirle, Philipp A. Schuster, Luis Kolb, Litwin Jacob, Alexander J.C. Kuehne

**Affiliations:** ^1^ Institute of Organic and Macromolecular Chemistry Ulm University Albert‐Einstein‐Allee 11 89081 Ulm Germany

**Keywords:** cycling stability, high‐capacity energy storage, organic batteries, redox‐active organic cathodes, π‐conjugated quinones

## Abstract

Sodium batteries (SBs) are a promising alternative to lithium‐ion batteries (LIBs) due to the abundance, cost‐effectiveness, and environmental sustainability of sodium. However, the larger ionic radius of Na^+^ leads to challenges in electrode stability, limiting the performance of conventional inorganic cathode materials. Redox‐active organic compounds, particularly π‐conjugated quinones, have emerged as a viable alternative, due to their tunable electrochemical properties, structural flexibility, and enhanced compatibility with Na^+^. Despite their advantages, many quinone‐based cathodes suffer from limited cycling stability and solubility issues. Here, the synthesis and characterization of poly(pentacenetetrone) (PPT) as a high‐capacity cathode material for SBs is reported. PPT exhibits a high theoretical specific capacity (*Q*_tsp_ = 319 mAh g⁻¹) and achieves an experimental specific capacity (*Q*_sp_) of 314 mAh g⁻¹ at 0.2C, with remarkable cycling stability. At 2C, the capacity remains at 260 mAh g⁻¹, retaining 92% after 500 cycles. PPT demonstrates excellent rate capability with 98% capacity retention after extended cycling. These findings highlight the potential of PPT as a high‐performance cathode material for sodium batteries, addressing critical challenges in scalability and long‐term stability for next‐generation energy storage systems.

## Graphical abstract

Poly(pentacenetetrone) (PPT) is introduced as a high‐capacity cathode for sodium batteries, offering 314 mAh g⁻¹ at 0.2C with excellent cycling stability. At 2C, it retains 92% capacity after 500 cycles. PPT's high performance and stability address key challenges in sodium battery technology, making it a promising material for next‐generation energy storage.

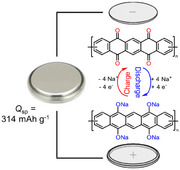

## 1 Introduction

Li‐ion batteries (LIBs) have shown great potential as a sustainable and reliable energy storage technology because of their high energy density and long cycle life. LIBs are utilized in various contemporary technologies, such as electric vehicles and portable electronics.^[^ ^1^, ^2^ ^]^ Nevertheless, the growing global energy demand will eventually surpass the Li resources needed for this technology. The limited availability and unequal distribution of lithium resources will increase its cost, which will further complicate the largescale use of LIBs in the future.^[^ ^3^, ^4^, ^5^ ^]^ In addition, geopolitical conflicts significantly limit the supply of rare electrode materials and lithium resources. This emphasizes the pressing need for high‐performance, reasonably priced energy storage systems that make use of earth‐abundant elements to produce a more sustainable battery technology. Sodium battery (SB) technology presents a promising alternative to LIB technology, given the natural abundance of sodium (2% of the Earth's crust), its high theoretical specific capacity (*Q*_tsp_ = 1165 mAh g^−1^), low cost, similar working principle but higher safety compared to LIBs.^[^ ^6^, ^7^, ^8^ ^]^ SBs offer a middle ground between the high performance of commercial LIBs and the goal of long‐term sustainability. While Li‐ and Na‐based battery technologies are transferable, the increased size of the Na^+^ ion (1.02 Å) compared to the Li^+^ ion (0.76 Å) causes a significant volume expansion during the sodiation process and irreversible phase change in the electrode materials typically employed for LIBs. As a consequence, the majority of conventional inorganic high‐capacity electrode materials suffer rapid capacity decay and battery failure, when directly transferred from LIB to SB technology.^[^ ^9^, ^10^ ^]^ Redox‐active organic compounds have emerged as promising candidates for cathodes in SBs, due to their structural tailorability, processability, fast kinetics, tunable and stable electrochemistry, and ability to accommodate Na^+^ ions. Unlike inorganic materials, organic redox materials are structurally flexible, due to their relatively weak intermolecular interactions. This flexibility entails less spatial hindrance during the reversible insertion and extraction of Na^+^ ions that guarantee high capacity and outstanding rate capability. Despite these advantages, only a few organic redox materials with high capacity and good cycling stability have been reported for SBs.^[^ ^11^, ^12^, ^13^, ^14^ ^]^  Several redox‐active groups, including phenoxyl, nitroxyl, viologen, carbazole, and quinone have been studied in various battery configurations.^[^ ^15^, ^16^, ^17^, ^18^, ^19^ ^]^ Among them, π‐conjugated quinones such as benzoquinones, naphthoquinones, and anthraquinones have emerged as stable redox materials of high theoretical specific capacity, redox reversibility, and resource availability.^[^ ^20^, ^21^, ^22^, ^23^, ^24^, ^25^, ^26^ ^]^ In these molecules, the π‐conjugation helps stabilize the charged states through delocalization, and when the π‐conjugation is extended, the planar quinone molecules stack on top of each other by π–π interactions to create layered structures with enhanced electrode characteristics, including high stability and fast charge transport.^[^ ^27^ ^]^ For instance, anthraquinone exhibits an improved cycling stability over benzoquinone.^[^ ^28^, ^29^ ^]^ However, its theoretical capacity is constrained by the additional aromatic units, which add molecular mass that does not contribute to the redox process. In addition, quinones are small organic molecules that dissolve in the electrolyte upon charging, which causes a rapid drop in capacity after just a few cycles. Hence, the challenge is to find a way to enhance the electrode capacity, while also allowing conjugation and increase of molecular mass to achieve the required stability.

**Figure 1:**
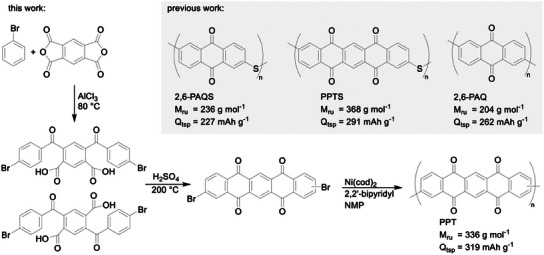
Quinone‐derived redox‐active polymers with the molar mass of the repeat unit (*M*_ru_) and theoretical specific capacity (*Q*_tsp_).^[^ ^27^, ^33^, ^35^ ^]^ Synthesis of PT via Friedel‐Crafts acylation and elimination of water. Polymerization to PPT is achieved by Yamamoto cross‐coupling.

Small molecular quinone molecules can be polymerized as a potent solution to the above‐described shortcomings. Polymerization of small redox molecules into a redox‐active homopolymer improves conjugation and retains the theoretical capacity per monomer unit. In addition, polymerization also improves the redox potential and leads to a reduction of the LUMO energy, as compared to the monomer.^[^ ^30^ ^]^ According to extended Flory‐Huggins solution theory, such redox polymers are highly robust and resistant to dissolution upon charging.^[^ ^31^, ^32^ ^]^ A number of differently connected poly(anthraquinones) with various backbone conjugations have been reported in recent years, and they show exceptional electrode characteristics in terms of stability and battery performance.^[^ ^33^ ^]^ One strategy to further improve the capacity of the redox active polymer is to replace the quinone repeating unit by pentacenetetrone (PT). In PT, the number of redox active quinone units is doubled, while the molecular weight only increases by about a third. However, this polymer remains inaccessible to date, due to synthetic limitations. The high potential of such material becomes apparent when consulting recent work investigating poly(pentacenetetrone sulfide) (PPTS).^[^ ^27^ ^]^ While the theoretical specific capacity of PPTS (*Q*_tsp_ = 291 mAh g^−1^) is promising, it only provides a slight advantage over the corresponding 2,6‐poly(anthraquinone) (2,6‐PAQ, *Q*_tsp_ = 262 mAh g^−1^) (see **Figure** **1**).^[^ ^34^ ^]^ Comparing 2,6‐PAQ with the recently reported poly(anthraquinone sulfide) (*Q*_tsp_ = 227 mAh g^−1^), it becomes evident that the redox‐inactive sulfide bridge in the polymer backbone limits the theoretical specific capacity (see Figure 1).^[^ ^35^ ^]^ The pentacenetetrone homopolymer – devoid of any redox‐inactive bridges – would instead provide a significant capacity increase to *Q*_tsp_ = 319 mAh g^−1^.

In this work, we present the synthesis and evaluation of poly(pentacenetetrone) (PPT) homopolymer as a high‐capacity cathode material for SBs. We efficiently prepare PPT from the pentacenetetrone monomer, dibrominated in the 2‐ and 9‐ or 10‐positions. We produce electrodes from PPT to characterize their performance as cathodes in SBs. Such a PPT‐based SB cell exhibits a capacity of *Q*_sp_ = 314 mAh g^−1^ at a rate of 0.2C, close to the theoretical limit. Moreover, when the cell is cycled at 2C, it delivers a stable capacity of 260 mAh g⁻^1^ after the 40th cycle and retains 92% of this capacity over the course of 500 cycles. Furthermore, the cell demonstrates an excellent rate capability by exhibiting 70 mAh g^−1^ at 50C and 251 mAh g^−1^ upon coming back to 2C, thus exhibiting negligible memory effect. As such, PPT represents an interesting material with excellent performance due to strong π‐interaction between the electrode material and the ions, high *Q*_tsp_ of 319 mAh g^−1^, and structural stability leading to stable capacity during extended cycling.

## 2 Results and Discussion

### 2.1 PPT Synthesis and Characterization

To synthesize a polymerizable PT monomer, we modify a previously reported protocol for dichloro‐5,7,12,14‐pentacenetetrone.^[^ ^36^ ^]^ Our synthesis makes use of a classical Friedel‐Crafts acylation between bromo‐benzene and pyromellitic dianhydride. Similar to the standard PT synthesis with benzene, this facilitates the opening of the anhydride, yielding two isomeric dicarboxylic acid intermediates (2,5‐dibenzoylterephthalic acid and 4,6‐dibenzoylisophthalic acid) (see Figure 1). Heating these acid intermediates in concentrated sulfuric acid facilitates the elimination of water and the closing of the aromatic ring system, yielding 2,9‐ or 2,10‐dibromo‐PT monomers (see Figure 1).^[^ ^37^ ^]^ The PPT homopolymer is obtained through Yamamoto coupling of these two isomeric dibromo‐PT monomers. The polymerization procedure is analogous to that of 2,6‐PAQ.^[^ ^38^, ^39^ ^]^ Unfortunately, the degree of polymerization in PPT could not be ascertained because of its insolubility in organic solvents. However, we are able to apply solid state ^13^C‐NMR, where we observe a small shift in the broadened polymer signals compared to the monomer indicating successful polymerization via cross‐coupling (see Figures S3 and S4, Supporting Information). In both spectra, the carbonyl signals in the downfield are clearly separated. The other carbon signals overlap to give a broad band between 120 and 140 ppm, which confirms the strong aromaticity of the PT unit.

The Fourier transform infrared spectra of PPT show a strong absorption peak at 1673 cm^–^¹ corresponding to the carbonyl (C═O) stretching vibrations, which remain unchanged from the monomer. But the change from monomer to polymer is visible in the vibrations of the aromatics at 1250 and 700 cm^–^^1^ (see Figure S5, Supporting Information). In addition, the thermogravimetric analysis (TGA) confirms good thermal stability for the PPT up to 300 °C, upon which decomposition over a broad temperature range occurs, indicating a dispersed polymer sample as expected from Yamamoto‐type polycondensation reaction (see Figure S6, Supporting Information). MALDI ToF analysis of the polymer corroborates the dispersity. All oligomers up to the hexamer can be detected; however, unfortunately, we do not obtain any information about the maximum chain length or the distribution. MALDI ToF confirms that there are bromine end‐groups, indicating that the coupling reaction is not efficient, probably due to the poor solubility and precipitation of polymer chains during the reaction (see Figure S8, Supporting Information).

Since our polymer is made from two isomeric monomers, the chains are not connected in a straight rigid rod‐like fashion but rather the structure of the solid polymer remains amorphous. This amorphous character is corroborated by the broad XRD signal, with absent crystallinity and only a few sharp peaks, which can be assigned to distances within the chains. The fine polymer particles that precipitate irregularly during the reaction show an amorphous morphology during inspection with scanning electron microscopy (SEM), further substantiating the hypothesis of a dispersed polymer sample (see Figure S9, Supporting Information).

### 2.2 Electrochemical Performance

**Figure 2:**
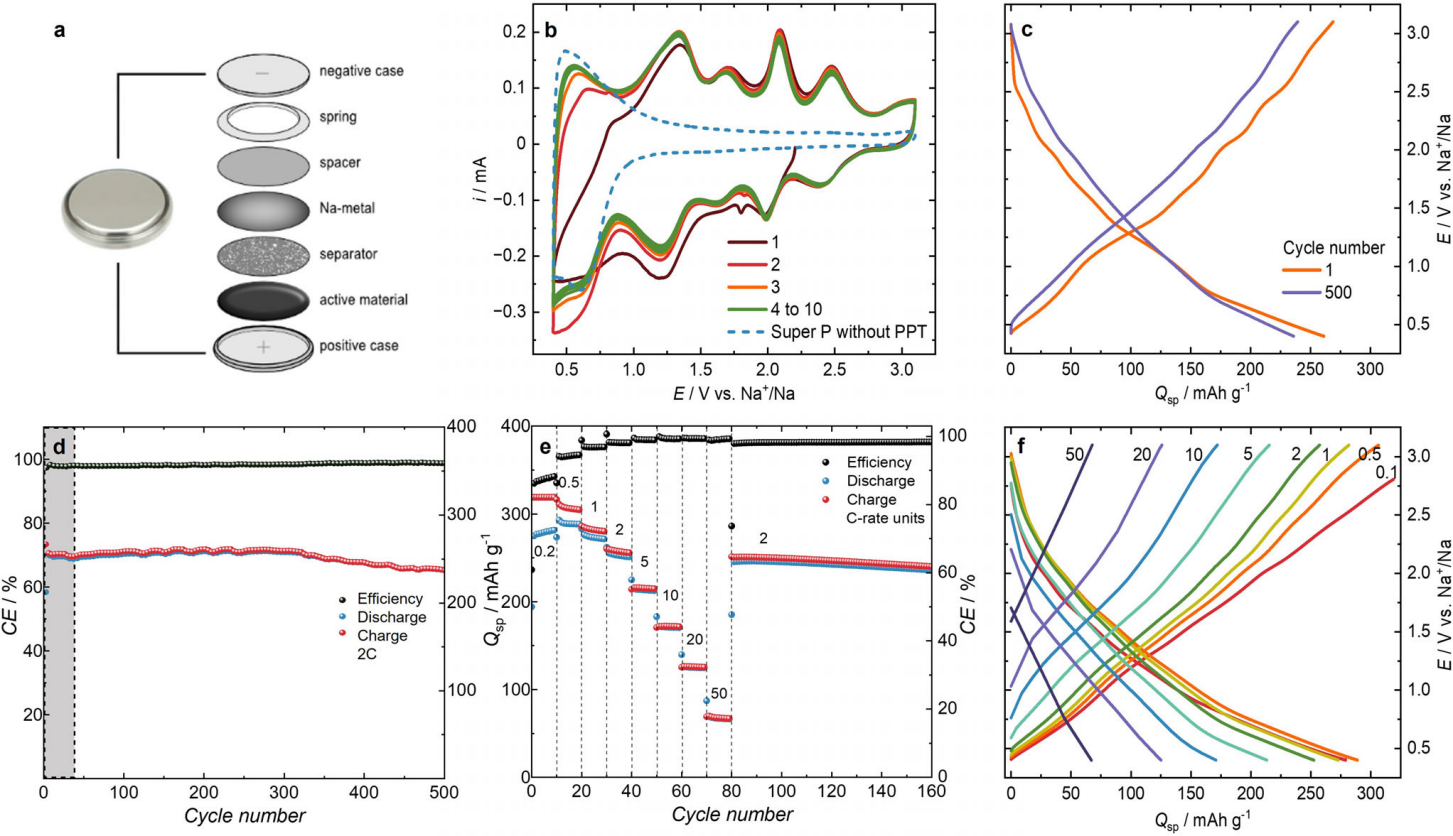
a) Schematic representation of the fabrication of the CR2032 Na half‐cell with a PPT composite cathode, b) potentiostatic cyclic voltammogram of the Na half‐cell of PPT (first 10 cycles) as a reference we show the Na half‐cell of Super P (blue dotted line), c) galvanostatic charge‐discharge measurements represented as the specific capacity versus the cell voltage (1^st^ and 500^th^ cycle), d) cycling performance of 500 cycles represented with efficiency and specific capacity versus cycle number operated at 2C rate, e) rate capability performance plotted as the specific capacity and coulombic efficiency versus the cycle number, f) cell voltage versus specific capacity for charge and discharge rates of 0.2, 0.5, 1, 2, 5, 10, 20, and 50C.

To investigate the electrochemical performance, we fabricate electrodes of our synthesized PPT by compounding with Super P as a conductive carbon additive and PVDF binder in the weight ratio of 45:45:10 with NMP as the solvent. This is a typical ratio employed for the majority of organic redox polymer electrodes in organic batteries.^[^ ^40^, ^41^, ^42^ ^]^ The resulting slurry is coated on to aluminum foil to obtain the cathode (see experimental section for comprehensive step‐by‐step manufacturing instructions and see Figure S11, Supporting Information). Coin cell fabrication involves placing the coated composite PPT electrode on the positive end plate, followed by a Whatman GF/D separator, and then 120 µL of 1 m NaPF_6_ in TEGDME electrolyte is added to wet the separator and the cathode. Subsequently, Na anode foil is placed on the separator, and then a stainless‐steel spacer, spring, and negative end plate are added. Finally, the stack is compacted with a crimper to obtain the SB coin cell, as shown in **Figure** **2**a. Carbonate based electrolytes are known to form unstable SEIs in SBs, leading to poor cycling performance. Glymes are a suitable alternative to carbonate‐based electrolytes as they are electrochemically stable and TEGDME possesses a high coordination number with Na^+^. Moreover, NaPF_6_ does not corrode Al surfaces, thereby enabling minimal chemical interference with the cell components during cell operation. Careful consideration of all these factors yields 1 m NaPF_6_ in TEGDME as our electrolyte system of choice (see Supporting Information for a more detailed rationale).

The freshly fabricated cell shows an open circuit voltage of ≈2.4 V. The compacted coin cell is allowed to rest for 3 h before the start of electrochemical studies. A similar procedure of fabrication has also been followed to fabricate cells with Li as the anode and 1 m LiPF_6_ in TEGDME as the electrolyte. After the cell fabrication, the half‐cells are subjected to potentiostatic cyclic voltammetry (CV) and galvanostatic charge‐discharge measurements, as shown in Figure S14 (Supporting Information). The Li cell displays a better capacity of 308 mAh g^−1^ at 1C. However, the cell loses 70% of the initial capacity to drop to 91 mAh g^−1^ by the 100^th^ cycle, indicating that PPT is better suited for Na than for Li cell operation. Furthermore, the SB cell is subjected to potentiostatic CV at 1 mV s^−1^ in the potential window of 0.4 to 3.1 V for 10 cycles, as seen in Figure 2b. In the first cathodic scan (reduction), the CV shows four prominent waves. In the subsequent scans, there are five oxidation and reduction waves centered ≈0.5, 1.3, 1.7, 2.0, and 2.4 V. From the PT repeat unit, a maximum of four redox events are to be expected. To explain the additional fifth wave in the CV, we hypothesize that the Super P conductive carbon might contribute to the redox behavior of the SB cell. To test this premise, we fabricate a new cell without PPT, using only Super P and PVDF on the cathode side (see Supporting Information for detailed manufacturing protocol). The CV of this PPT‐free reference cell shows a single redox wave ≈0.5 V, substantiating that this wave does not belong to PPT but comes from the other electrode components (see Figure 2b). Previously, the effect of natural graphite on the capacity of battery cells has been studied in ether‐based solvents. Here as well, Na^+^ ions intercalate into natural graphite, contributing to the overall capacity.^[^ ^43^ ^]^ Generally, the co‐intercalation potentials of Na^+^ with solvent are in the potential range of 0.5–1 V, which is also the range where we observe the additional peak in our work.^[^ ^44^ ^]^ So, we presume that the additional peak that we observe ≈0.5 V is due to co‐intercalation of Na^+^ ions along with solvent molecules into the Super P carbon. Interestingly, this wave occurs at a slightly higher potential in the PPT‐based cell and moves gradually to 0.5 V during the first three cycles, whereas the reduction event does not shift (see the discussion for the contribution of Super P to the total cell capacity in the Supporting Information, see also Figure S15, Supporting Information). The kinetics of oxidation (desodiation) improve as Super P becomes gradually more sodiated during the first three cycles, after which the carbon will have expanded and reached equilibrium. The remaining four waves in the CV can now be clearly assigned to the PPT, with each quinone group contributing one individual single electron redox event (see Scheme S1, Supporting Information for a proposed scheme of charge/discharging on the molecular scale). To further understand the effect of PT polymerization, we fabricate a cell using the PT monomer. CV measurements at 1 mV s⁻¹ with the PT composite as the cathode show a significant current drop by the fifth cycle (see Figure S12, Supporting Information). This drop in current will possibly be due to the significant dissolution of PT in the electrolyte medium, as seen in Figure S13 (Supporting Information). Charge‐discharge analysis at 2C reveals minimal capacity utilization and lower discharge voltage. These results clearly demonstrate that polymerization effectively reduces the dissolution of the active material and increases its redox potential by stabilizing the LUMO.^[^ ^30^ ^]^

To learn more about the charging and discharging behavior of our PPT‐based SB cell, we perform galvanostatic charge‐discharge experiments at 2C (1C is 319 mA g^−1^). The SB cell shows an initial capacity of 268 mAh g^−1^ in the first cycle (see Figure 2c). Over the next 20 cycles, the capacity drops to 255 mAh g^−1^, only to improve again to 260 mAh g^−1^, where the cell stabilizes after 40 cycles with the coulombic efficiency (CE) of the cell approaching 99.9% (see Figure 2d). We consider this as the conditioning period for our batteries, which indicates the combined effect of the formation of a stable SEI and the creation of diffusion pathways for the Na^+^ ions.^[^ ^45^ ^]^ By the end of 500 cycles, the cell shows a capacity of 241 mAh g^−1^, retaining 92% of the capacity after conditioning (see Figure 2c,d). Hence, the cell shows a low rate of fading of 0.01% per cycle. Similarly, cycling studies are carried out at 1C current rating with the cell exhibiting an initial capacity of 286 mAh g^−1^ with a CE of 98%, as seen in Figure S16a,b (Supporting Information). The capacity drops to 280 mAh g^−1^ in the next 10 cycles and improves to 285 mAh g^−1^ by the 30^th^ cycle and stabilizes, similar to the conditioning period in our cell at 2C. The cell shows a capacity of 245 mAh g^−1^ by the end of 500 cycles, thus retaining 86% of its initial capacity (with a fading rate of 0.03% per cycle). To evaluate the electrochemical stability of PPT, we perform charge‐discharge cycling at a low current of 0.15C. The cell initially loses capacity but gradually recovers after 15 cycles, as shown in Figure S17 (Supporting Information). This capacity increase likely results from the polymer's amorphous nature, which enables chain reorganization and the formation of low‐energy diffusion pathways, enhancing stability.

**Figure 3:**
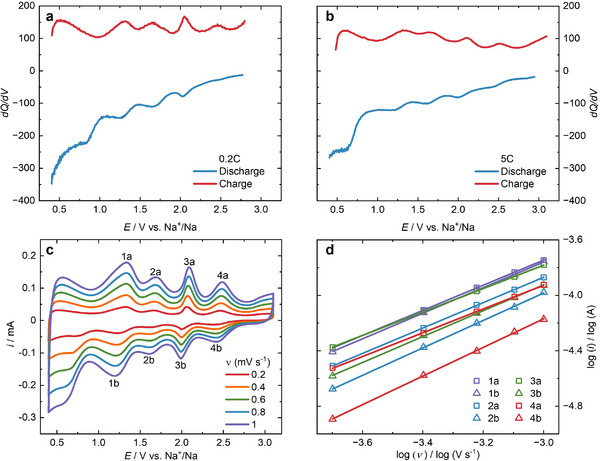
DCA plots of *dQ/dV* versus *E* versus Na^+^/Na to understand the faradaic behavior of the cell during galvanostatic charge‐discharge experiments at a) 0.2C and b) 5C (chosen from the rate capability study). c) Plot of current versus potential for the CV at different scan rates of 0.2, 0.4, 0.6, 0.8, and 1.0 mV s^−1^, d) log‐log plot showing the linear behavior of the absolute peak current versus the scan rate.

Notably, the cell maintains its capacity without fading even after 120 days of cycling, demonstrating the polymer's strong electrochemical performance. A close comparison of the capacities of the cells at 1C and 2C reveals that the capacity loss due to ohmic resistance in the cell is minimal. This prompts us to carry out rate capability studies for our PPT‐based SB at the current ratings of 0.2, 0.5, 1, 2, 5, 10, 20, and 50C, as shown in Figure 2e,f. The cell shows a capacity of 318 mAh g^−1^ at the initial 0.2 C‐rate and subsequently, the current is adjusted after every 10 cycles. The cell exhibits a capacity of 255 mAh g^−1^ at 2C (after 40 cycles). At the highest rate of charging and discharging, 50C, the cell showed a capacity of 70 mAh g^−1^. Upon coming back to 2C (by the 81^st^ cycle), the cell exhibits a capacity of 251 mAh g^−1^, translating to a capacity retention of 98% (compared to 255 mAh g^−1^ in the 40^th^ cycle). Charge‐discharge at low C‐rate would take longer to complete for the single charge and discharge cycle and hence the probability of parasitic side reactions is higher. Consequently, the expected CE would be moderate at low C‐rate with CE being 88.2% at 0.2C and rising as the C‐rate is increased. For a detailed parametric comparison of the electrochemical performance of PPT with materials used in SBs, the reader is referred to Table S3 (Supporting Information) in the Supporting Information. Interestingly, the SB cell does not show any memory effects during the rate capability study. The ability of the composite to retain its capacity even after subjecting to high current variation (from 0.2 to 50 C and back to 2 C) is surprising. Moreover, we do not observe a distinct charge‐discharge plateau, as the charge is delocalized within the extended π‐system of the polymer.^[^ ^46^ ^]^ Additionally, the redox‐active centers exhibit minimal potential difference, leading to an almost continuous charge‐discharge profile without well‐defined plateau regions. This behavior also suggests the presence of capacitive effects alongside the Faradaic contribution to the capacity of the cell. To deconvolute faradaic from capacitive contributions to the capacity exhibited by PPT electrodes, we perform differential capacity analysis (DCA) by plotting the first derivative *dQ*/*dV* against the cell voltage (see **Figure** **3**a). DCA is an effective tool for distinguishing redox events during charging and discharging, separating faradaic processes from capacitive behavior and kinetic overpotential effects. From the CV it is evident that our PPT redox polymer exhibits narrowly spaced and therefore inseparable redox peaks at 1.3, 1.7, 2.0, and 2.4 V, complicating the deconvolution of capacitive from faradaic processes toward to total capacity of the cell. DCA analysis at 0.2 and 5C rates provide qualitative insights into the faradaic contribution to the total cell capacity (see Figure 3a,b). The DCA results clearly demonstrate that PPT offers exceptional reversibility, as evidenced by the minimal overpotential separation between its oxidation and reduction peaks. While DCA confirms some faradaic contribution, the absence of well‐defined peaks at high C‐rates indicates that the high capacity of the PPT cell is more strongly dominated by non‐faradaic processes, in line with the charge‐discharge analysis (see Figure 2f). The minimal separation between oxidation and reduction peaks is also a clear indication that the cell offers low solution resistance (R_S_) and excellent diffusion of Na^+^ ions supporting our understanding of the cell with its excellent reversibility and rate capability.

Following this, we perform electrochemical impedance spectroscopy (EIS) prior to and after 10 cycles of CV (see Figure S19, Supporting Information). The results indicate a low and identical R_S_ of 26.2 and 26.1 Ω before and after CV cycling. However, the charge transfer resistance (R_CT_) drops from 76.4 to 50.7 Ω under the same experimental conditions. This drop in R_CT_ upon cycling emphasizes our hypothesis wherein a few initial cycles are attributed to the conditioning of the cell, due to SEI layer formation and the creation of diffusion paths for the subsequent cycling.^[^ ^45^ ^]^ The Nyquist plot shows a 45° slope in the low frequency region indicating Warburg impedance, characteristic of diffusion limited charge‐transfer. To understand the role of Na^+^ ion diffusion in our SB, we recorded CV at different scan rates of 0.2, 0.4, 0.6, 0.8, and 1.0 mV s^−1^, as shown in Figure 3c. As we increase the scan rate, the current increases, while the oxidation peaks shift to higher potentials, whereas the reduction peaks shift to lower ones. However, the shifts in potential are minimal, substantiating the low ohmic polarization of the cell characterized by low R_S_ as determined by EIS in Figure S19 (Supporting Information). The CVs show reversible redox behavior, which can be analyzed according to Equation (1),

(1) logi=a+blog(ν)

where *a* and *b* are variable parameters with slope *b* varying from 0.5 to 1, *i* as the measured current in A, and *ν* is the scan rate in V s^−1^. When the slope *b* is close to 0.5, a semi‐infinite diffusion‐controlled behavior can be assumed, while when it is close to 1.0, it is capacity‐controlled. The slope values for our four oxidation and reduction events in PPT are close to 1 (Table S1, Supporting Information), indicating that the charge‐discharge processes are predominantly capacitive in nature (see Figure 3d). Additionally, the capacitive and faradaic contributions are additive in nature and can be estimated from Equation (2),

(2) $$i=k_{1}\nu +k_{2}\nu^{1/2}$$

where *i* is the measured current in A, *k*_1,_ and *k*_2_ are constants, and *ν* is the scan rate in V s^−1^. The term *k*_1_ *ν* represents the capacitive contribution, and *k*_2_ *ν* ^1/2^ represents the faradaic contribution. From the log‐log plot of *i* versus *ν*, it becomes evident that the redox events are capacitive and faradaic in nature, and that the capacitive contribution increases as the scan rate increases (see the bar graph in Figure S20, Supporting Information). For a scan rate *ν* = 0.2 mV s^−1^, a substantial faradaic contribution of 26% to the overall capacity is observed, whereas this faradaic contribution is halved at 1 mV s^−1^. This indicates that surface confined charge transfer phenomena predominate at higher rates of charge‐discharge over diffusion‐controlled processes.

**Figure 4:**
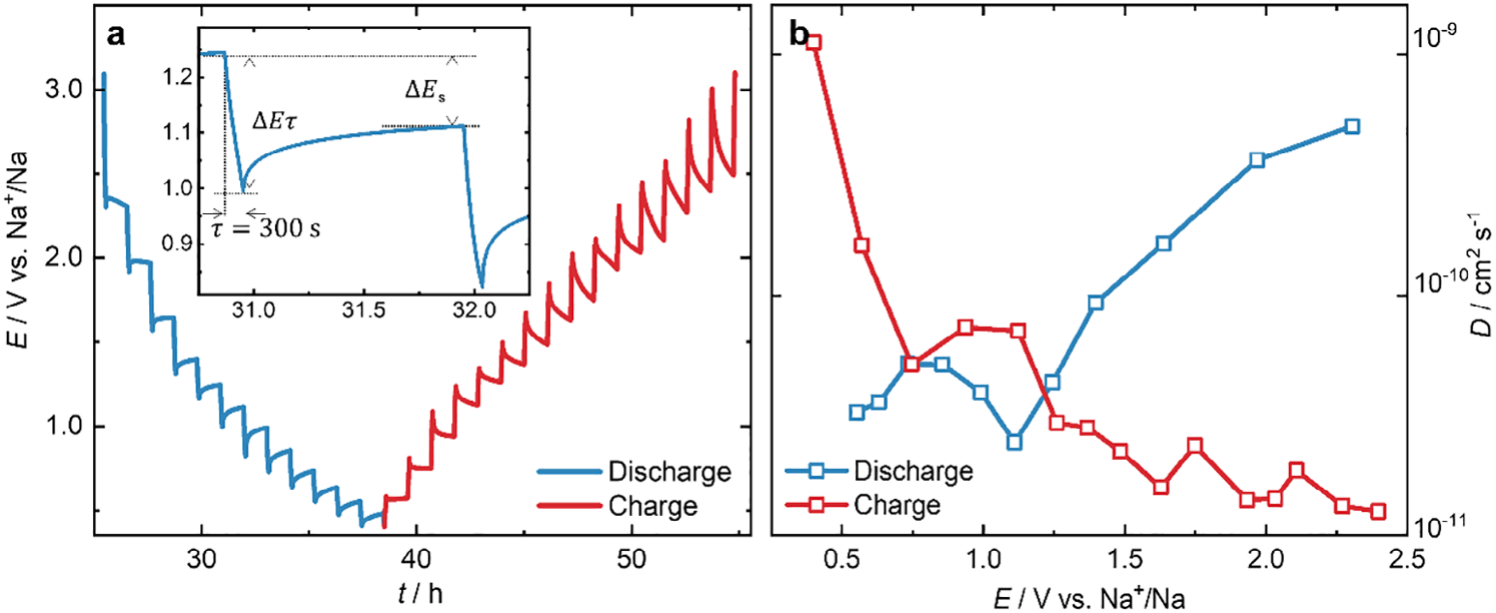
a) GITT plot of cell voltage versus time for one discharge step with minimal overvoltage loss (inset: GITT curve for one complete cycle), b) diffusion coefficient of Na^+^ ions at different cell voltage calculated from GITT.

To further understand the diffusion of Na^+^ ions, we perform galvanostatic intermittent titration (GITT). The diffusion coefficient (*D*) of Na^+^ ions over a voltage range is determined using Equation (3).^[^ ^27^, ^47^ ^]^

(3) $$D=\frac{4}{\pi \;\tau }{\left(\frac{m_{B}V_{M}}{M_{B}\;S}\right)}^{2}{\left(\frac{\Delta E_{S}}{\Delta E_{\tau }}\right)}^{2}$$

where *D* is the diffusion coefficient in cm^2^ s^−1^, *τ* is the pulse time, *m*_B_ is the mass loading, *V*_M_ is the molar volume and *M*_B_ is molar mass of the PPT repeat unit (PT), and *S* is the electrode‐electrolyte geometrical area. Δ*E*_S_ is the change in steady‐state (equilibrium) voltage upon application of a small current and Δ*E*_τ_ is the total change in voltage during the application of a current pulse, neglecting the internal resistance of the cell. The molar volume of the PT repeat unit is determined by measuring the weight of a pressed PPT pellet of precise dimensions (*V*_M_ = 337.9 cm^3^ mol^−1^), as shown in Figure S20 (Supporting Information). It is important to note that the *V*_M_ of PPT is similar to the value reported for small molecular PT crystals (*V*_M_ = 217.8 cm^3^ mol^−1^).^[^ ^48^ ^]^ We note, that the determination of *V*_M_ using a pellet can lead to overestimation, as the pressed pellet may not be as dense as in the case of a crystal. From the GITT analysis, the diffusion coefficient is estimated to be ≈1 × 10^−10^ cm^2^ s^−1^, which is in agreement with the *D* coefficients for other polymers and inorganic molecules compiled in Table S2 (Supporting Information) (see **Figure** **4**a,b). It is important to note that the *D* values for organic materials are a few orders of magnitude higher than *D* values of inorganic redox materials, as diffusing ions experience less electrostatic repulsion in an organic material than in an inorganic material (summarized in Table S2, Supporting Information). The combination of low internal resistance, π‐interaction, higher capacitive contribution, and better *D* leads to higher charge‐discharge rates and an improved rate capability in our organic PPT electrode.

To support our claim that the PPT‐based cell exhibits Faradaic behavior, we perform X‐ray photoelectron spectroscopy (XPS) to gain deeper insight into the charge storage mechanism. We conduct XPS measurements on the pristine electrode and on electrodes after the 10^th^ discharge and charge cycle. The detailed C 1s spectra show significant contributions of aromatic C═C, C═O, and C─O from PPT, alongside aliphatic C─C/C─H and a minor contribution from O─C═O, which will originate from the PVDF binder and conductive carbon. Our C 1s spectral analysis reveals that C═O contributes 4.77% in the pristine state, 2.40% in the discharged state, and 3.84% in the charged state, as shown in Figure S22 (Supporting Information). This decrease in C═O bonding during discharge suggests its involvement in the redox process. Meanwhile, the contribution of C─O bonding increases from 6.27% in the pristine state to 8.03% in the discharged state before decreasing to 5.60% upon charging. These transformations indicate that C═O groups in the pristine state convert to C─O⁻ during discharge and revert to C═O upon charging. This cyclic transformation confirms the redox activity of the quinone moieties in the charge‐discharge process.

## 3 Conclusions and Perspectives

In this work, we design and synthesize a π‐conjugated redox‐active PPT homopolymer through a halogenation strategy and demonstrate its potential as a high‐capacity cathode material for SBs. We describe its excellent rate capability, which is due to a combination of low internal resistance, high capacitive contribution, and high *D*. These performance metrics suggest that the PPT is suitable for high‐power applications. PPT is a versatile cathode material, which allows it to be used in different metal batteries. In the future, the performance of this material could be expanded to other metals by functionalization with electron withdrawing atoms, which reduces the LUMO level and enhances the potential of the electrode without compromising the *Q*_tsp_.

## 4 Experimental Section

### Electrode Preparation

The synthesized PPT was combined with Super P conductive additive and poly(vinylidene fluoride) (PVDF) binder in a ratio of 45:45:10 (wt.%) and thoroughly ground for 20 min using a mortar and pestle to form a fine composite, as schematically illustrated in Figure S11 (Supporting Information). The composite was transferred into a Thinky mixer capsule, and a few drops of *N*‐methyl‐2‐pyrrolidone (NMP) solvent were added. The capsule was then subjected to centrifugation for 1 h to obtain a uniform slurry of the composite. The resulting slurry was cast onto aluminum foil using the doctor blade technique and dried at 80 °C overnight to achieve a uniformly coated layer. The coated foil was then cut into circular electrodes with a diameter of 12 mm using a precision cutter. To remove any residual moisture, the cut electrodes were vacuum‐dried at 120 °C for 3 h. Subsequently, the dried electrodes and drying apparatus were transferred into an argon‐filled glove box with oxygen and moisture levels maintained at ≤ 0.1 ppm. CR2032 coin cells were then fabricated using the prepared electrodes. The procedure was repeated using PPT:Super P:PVDF in an 80:15:5 ratio to fabricate cathodes containing 80% active material. These cathodes were utilized to evaluate the performance of higher active material loading and for XPS analysis.

### Electrochemical Measurements

CR2032 half‐cells were assembled inside a glove box maintained at < 0.1 ppm for H_2_O and O_2_. Na metal was purchased from Sigma‐Aldrich, rolled into foil, and used as the counter electrode. GF/D Whatmann glass fiber filter paper was used as a separator. 1 m NaPF_6_ in TEGDME purchased from E‐Lyte was used as an electrolyte without further purification. The electrode had a uniform coating of 0.8 to 1 mg of the composite in 1 cm^2^ area. The specific capacity of each cell was calculated based on the mass of the active material. For the cell used for calculating capacity contribution from Super P, mass of the Super P alone was considered. The cells were allowed to rest for 3 h before starting electrochemical operations. Cells used for 1C, 2C and rate capability studies were conditioned by operating the cells at 0.5C for 5 cycles before using it for further studies. All the galvanostatic studies were carried out with VMP electrochemical workstation maintained at 23 °C temperature. EIS was recorded from 10 kHz to 100 mHz with a potential perturbation of 10 mV at the open circuit voltage.

## Conflict of Interest

The authors declare no conflict of interest.

## Supporting information



Supporting Information

## Data Availability

The data that support the findings of this study are available from the corresponding author upon reasonable request.

## References

[1] J. B. Goodenough , K. S. Park , J. Am. Chem. Soc. 2013, 135, 1167. 23294028 10.1021/ja3091438

[2] M. Li , J. Lu , Z. Chen , K. Amine , Adv. Mater. 2018, 30, 1800561. 10.1002/adma.201800561 29904941

[3] P. H. Camargos , P. H. J. dos Santos , I. R. dos Santos , G. S. Ribeiro , R. E. Caetano , Int. J. Energy Res. 2022, 46, 19258.

[4] A. Luntz , J. Phys. Chem. Lett. 2015, 6, 300. 26263466 10.1021/jz502665r

[5] J. W. Choi , D. Aurbach , Nat. Rev. Mater. 2016, 1, 16013.

[6] J. Chen , G. Adit , L. Li , Y. Zhang , D. H. C. Chua , P. S. Lee , Energy & Environmental Materials. 2023, 6, 12633.

[7] J.‐Y. Hwang , S.‐T. Myung , Y.‐K. Sun , Chem. Soc. Rev. 2017, 46, 3529. 28349134 10.1039/c6cs00776g

[8] C. Delmas , Adv. Energy Mater. 2018, 8, 1703137.

[9] A. Manthiram , Nat. Commun. 2020, 11, 1550. 32214093 10.1038/s41467-020-15355-0 PMC7096394

[10] K. M. Abraham , ACS Energy Lett. 2020, 5, 3544.

[11] H. Zhang , Y. Gao , X. Liu , L. Zhou , J. Li , Y. Xiao , J. Peng , J. Wang , S. Chou , Adv. Energy Mater. 2023, 13, 2300149.

[12] Y. Lu , Q. Zhang , L. Li , Z. Niu , J. Chen , Chem. 2018, 4, 2786.

[13] T. B. Schon , B. T. McAllister , P.‐F. Li , D. S. Seferos , Chem. Soc. Rev. 2016, 45, 6345. 27273252 10.1039/c6cs00173d

[14] Y. Lu , J. Chen , Nat. Rev. Chem. 2020, 4, 127. 37128020 10.1038/s41570-020-0160-9

[15] C. R. Mirle , M. Raja , P. Vasudevarao , S. Sankararaman , R. Kothandaraman , New J. Chem. 2020, 44, 14401.

[16] Y. Wu , R. Zeng , J. Nan , D. Shu , Y. Qiu , S. L. Chou , Adv. Energy Mater. 2017, 7, 1700278.

[17] T. Liu , X. Wei , Z. Nie , V. Sprenkle , W. Wang , Adv. Energy Mater. 2016, 6, 1501449.

[18] A. Orita , M. G. Verde , M. Sakai , Y. S. Meng , J. Power Sources. 2016, 321, 126.

[19] T. Jähnert , M. D. Hager , U. S. Schubert , Macromol. Rapid Commun. 2016, 37, 725. 26937847 10.1002/marc.201500702

[20] Y. Hanyu , Y. Ganbe , I. Honma , J. Power Sources. 2013, 221, 186.

[21] M. Lee , J. Hong , H. Kim , H. Lim , S. B. Cho , K. Kang , C. B. Park , Adv. Mater. 2014, 26, 2558. 24488928 10.1002/adma.201305005

[22] X. Zhou , R. A. J. Janssen , S. Er , Energy Advances. 2023, 2, 820. 37323160 10.1039/d2ya00282e PMC10267898

[23] C. Wang , C. Jiang , Y. Xu , L. Liang , M. Zhou , J. Jiang , S. Singh , H. Zhao , A. Schober , Y. Lei , Adv. Mater. 2016, 28, 9182. 27571544 10.1002/adma.201603240

[24] M. Yao , H. Senoh , T. Sakai , T. Kiyobayashi , Int. J. Electrochem. Sci. 2011, 6, 2905.

[25] R. Russo , C. Davoisne , A. Urrutia , Y. Danten , C. Gatti , G. Toussaint , P. Stevens , C. Frayret , M. Becuwe , ACS Appl. Polym. Mater 2023, 5, 9865.

[26] V. Pignier , S. Toumieux , C. Davoisne , M. Caroff , A. Jamali , S. Pilard , D. Mathiron , D. Cailleu , F. Delattre , D. P. Singh , R. Douali , M. Becuwe , Small. 2024, 20, 2305701. 10.1002/smll.202305701 37712120

[27] M. Tang , S. Zhu , Z. Liu , C. Jiang , Y. Wu , H. Li , B. Wang , E. Wang , J. Ma , C. Wang , Chem. 2018, 4, 2600.

[28] Z. Song , H. Zhan , Y. Zhou , Chem. Commun. 2009, 4, 448. 10.1039/b814515f 19137181

[29] T. Yokoji , H. Matsubara , M. Satoh , J Mater Chem A Mater 2014, 2, 19347.

[30] B. Flamme , B. Jismy , M. Abarbri , M. Anouti , Mater. Adv. 2021, 2, 376.

[31] P. J. Flory , J. Chem. Phys. 1953, 21, 162.

[32] M. Muthukumar , Macromolecules. 2017, 50, 9528. 29296029 10.1021/acs.macromol.7b01929 PMC5746850

[33] Z. Song , Y. Qian , M. L. Gordin , D. Tang , T. Xu , M. Otani , H. Zhan , H. Zhou , D. Wang , Angew. Chem. 2015, 127, 14153. 10.1002/anie.201506673 26411505

[34] W. Mao , Y. Ding , M. Li , C. Ma , Z. Cao , C. He , K. Bao , Y. Qian , ChemElectroChem. 2021, 8, 1678.

[35] I. Gomez , O. Leonet , J. Alberto Blazquez , H.‐J. Grande , D. Mecerreyes , ACS Macro Lett. 2018, 7, 419. 35619336 10.1021/acsmacrolett.8b00154

[36] E. Philippi , F. Auslaender , Monatsh. Chem. 1921, 42, 1.

[37] E. Philippi , Monatsh. Chem. 1911, 1, 631.

[38] T. Yamamoto , H. Etori , Macromolecules. 1995, 28, 3371.

[39] D. Wielend , Y. Salinas , F. Mayr , M. Bechmann , C. Yumusak , H. Neugebauer , O. Brüggemann , N. S. Sariciftci , ChemElectroChem. 2021, 8, 4360.

[40] J. Hu , Y. Hong , M. Guo , Y. Hu , W. Tang , S. Xu , S. Jia , B. Wei , S. Liu , C. Fan , Q. Zhang , Energy Storage Mater. 2023, 56, 267.

[41] W. Huang , M. Zhang , H. Cui , B. Yan , Y. Liu , Q. Zhang , Chem Asian J. 2019, 14, 4164. 31654601 10.1002/asia.201901344

[42] T. Liu , K. C. Kim , B. Lee , Z. Chen , S. Noda , S. S. Jang , S. W. Lee , Energy Environ. Sci. 2017, 10, 205.

[43] H. Kim , J. Hong , Y. U. Park , J. Kim , I. Hwang , K. Kang , Adv. Funct. Mater. 2015, 25, 534.

[44] M. L. Divya , Y. S. Lee , V. Aravindan , ACS Energy Lett. 2021, 6, 4228.

[45] M. Chen , W. Hua , J. Xiao , D. Cortie , X. Guo , E. Wang , Q. Gu , Z. Hu , S. Indris , X. Wang , Angew. Chem. 2020, 132, 2470. 10.1002/anie.201912964 31657087

[46] S. Muench , A. Wild , C. Friebe , B. Häupler , T. Janoschka , U. S. Schubert , Chem. Rev. 2016, 116, 9438. 27479607 10.1021/acs.chemrev.6b00070

[47] W. Weppner , R. A. Huggins , J. Electrochem. Soc. 1977, 124, 87.

[48] D. Käfer , M. El Helou , C. Gemel , G. Witte , Cryst. Growth Des. 2008, 8, 3053.

