# Supplementary material for: Poly(pentacenetetrone) as a High‐capacity Cathode for Sodium Batteries
Source: Adv Sci (Weinh). 2025 Mar 26;12(19):2500484. doi: 10.1002/advs.202500484 (PMC12097111; doi:10.1002/advs.202500484)
Supplement: Supplementary file 1 — Supporting Information [file ADVS-12-2500484-s001.pdf]

ADVANCED SCIENCE
Open Access

Supporting Information

for *Adv. Sci.*, DOI 10.1002/advs.202500484

Poly(pentacenetetrone) as a High-capacity Cathode for Sodium Batteries

*Chinmaya Mirle, Philipp A. Schuster, Luis Kolb, Litwin Jacob and Alexander J.C. Kuehne**

Supporting Information

Poly(pentacenetetrone) as a High-capacity Cathode for Sodium Batteries

Chinmaya Mirle,[†] *Philipp A Schuster,*[†] *Luis Kolb, Litwin Jacob, and Alexander J.C. Kuehne* *

Institute of Organic and Macromolecular Chemistry

Ulm University, Albert-Einstein-Allee 11, 89081 Ulm, Germany

Table of Contents

1. General Methods and Instrumentation
2. Synthesis of 2,5-bis(4-bromobenzoyl)terephthalic acid and 2,4-bis(4-bromobenzoyl)terephthalic acid
3. Synthesis of 2,9-dibromopentacene 5,7,12,14-tetrone and 2,10-dibromopentacene 5,7,12,14-tetrone.
4. Synthesis of poly(pentacenetetrone).
5. Synthesis of 2,9-dichloropentacene 5,7,12,14-tetrone and 2,10-dichloropentacene 5,7,12,14-tetrone.
6. PPT free electrode preparation
7. Choice of suitable electrolyte
8. Electrochemical characterizations with schemes and figures

1. General Methods and Instrumentation

NMR spectroscopy

The ^{1}H-NMR spectra (referenced against the solvent peak of DMSO-d_6) are measured with an AMX 400 spectrometer from Bruker. The data analysis was carried out with the MestReNova software.

The ^{13}C-NMR solid state spectra were recorded with the Cross-Polarization Magic Angle Spinning (CPMAS) method on the same Bruker spectrometer.

TGA analysis

We use a TGA 8000™ thermogravimetric analyzer from PerkinElmer for the measurement. The measurement is performed under N_2 atmosphere with a heating rate of 10 °C/min.

Scanning Electron Microscopy (SEM)

The sample was finely dispersed and applied to a silicon wafer by drop casting. The sample was then sputter coated (Cressington 108auto/SE) with 5 nm Au/Pd and measured on an electron microscope (Hitachi S-2500) with 20 kV acceleration voltage. Images were analyzed using Fiji – ImageJ software.

Fourier transform infrared spectroscopy

FTIR spectroscopy was performed with a Perkin Elmer spectrometer, model Spectrum TWO.

Elemental analysis

Elementar vario Micro cube setup for the determination of C, H, N and S content.

Mass spectroscopy

We use a solvent free sample preparation with trans-2-[3-(4-tert-butylphenyl)-2-methyl-2-propenylidene]malononitrile (DCTB) as matrix on a Bruker MALDI-TOF/TOF (ultrafleXtreme) device (negative mode).

XRD

Amorphous nature of the polymer were analyzed using X-ray powder diffraction (XRD, Stoe STADI P XRD diffractometer) in Debye-Scherrer geometry using a Mo Kα X-ray source

(λ = 0.0709 nm) at 50 kV, 40 mA. Diffraction patterns were recorded in a 2θ angle range between 5 and 50°.

XPS

X-ray photoelectron spectroscopy (XPS) measurements were carried out on a Specs XPS system with a Phoibos 150 energy analyzer. Cells in their 10th discharge and charge were used for respective analysis. Cells were opened inside an Ar-filled glovebox with <0.1 ppm H_2O level, washed with TEGDME solvent and dried at 100 °C for 24 h before preparing the samples. The spectra were recorded using monochromatized Al Kα radiation (200 W, 12 kV) and pass energies 30 eV for detail measurements. All binding energies were calibrated to the C1s peak of C-H species at 285.0 eV. The peak fit of the XPS results was done with CasaXPS, using Shirley-type backgrounds and Gaussian-Lorentzian (GL30) peak shapes. For the pristine sample, two peaks with the same intensity were added, which account for the CH_2 (286.3 eV) and CF_2 (290.8 eV) groups of the PVDF binder.

2. Synthesis of 2,5-bis(4-bromobenzoyl)terephthalic acid and 2,4-bis(4-bromobenzoyl)terephthalic acid.

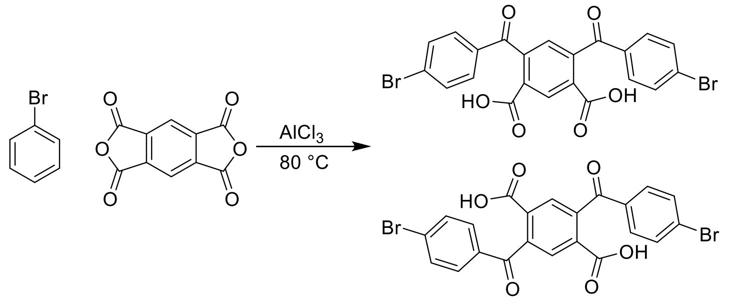

To a mixture of pyromellitic dianhydride (5 g, 22.9 mmol) and bromobenzene (50 mL, 478 mmol) heated to 80 °C in a dry flask, $AlCl_3$ (12.5 g, 93.7 mmol) was added in several small fractions.[1] The reaction mixture turns darker as the reaction progresses, and it is continued to stir at 80 °C for 12 h. The reaction mixture is then poured slowly into an ice-hydrochloric acid mixture and allowed to stir for 10 min. The precipitated brown solid is filtered and washed with excess water. The collected solid is dissolved in $NaHCO_3$ solution by stirring until no bubbles remain. The solution obtained is filtered through a filter paper, and then bromobenzene residues remain at the bottom of the aqueous solution. The solution is then washed twice with DCM or until the aqueous solution is colorless. Finally, the aqueous solution is acidified with hydrochloric acid, and the precipitated pure product is filtered and dried in an oven at 80 °C

to give the two isomers, 2,5-bis(4-bromobenzoyl)terephthalic acid and 2,4-bis(4-bromobenzoyl)terephthalic acid (66.5% yield for the mixture). IR: 3700 – 2800 cm^{-1} very broad ν(OH) carbon acid, 1750 – 1600 cm^{-1} broad ν(C=O) carbonyl, 1250 cm^{-1} broad δ(C-H) aromatic. ^{1}H-NMR (400 MHz, DMSO-d_6, 350 K): δ [ppm] = 8.56 (s, 2H), 7.97 – 7.91 (m, 2H), 7.80 – 7.45 (m, 8H).

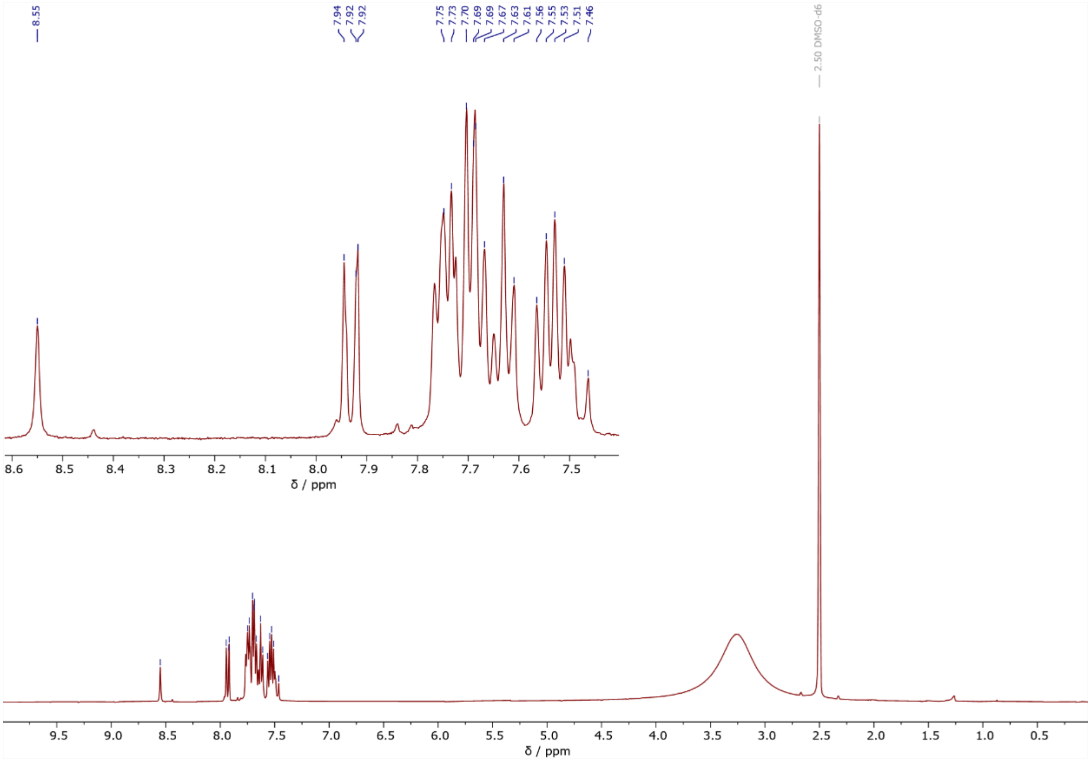

Figure S1 ^{1}H-NMR spectrum of the mixture 2,5-bis(4-bromobenzoyl)terephthalic acid and 2,4-bis(4-bromobenzoyl)terephthalic acid (400 MHz, DMSO-d_6, 350 K).

3. Synthesis of 2,9-dibromopentacene 5,7,12,14-tetrone and 2,10-dibromopentacene 5,7,12,14-tetrone.

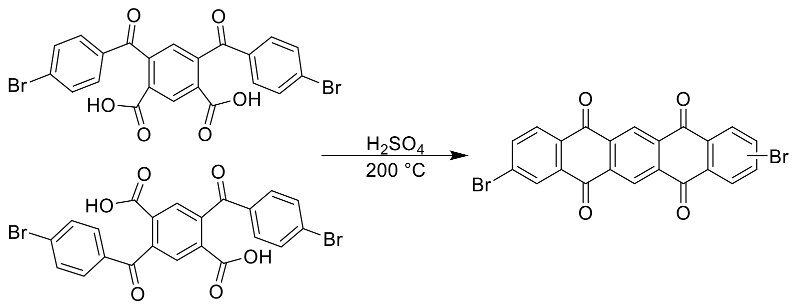

Mixture of isomers, 2,5-bis(4-bromobenzoyl)terephthalic acid and 2,4-bis(4-bromobenzoyl)terephthalic acid (2 g, 3.75 mmol) is heated in concentrated sulfuric acid (50

mL) at 100 °C for one hour, and then the reaction was continued at 200 °C for another hour. [2,3] Towards the end of the reaction, the solution becomes cloudy, and a yellow solid precipitates out. After cooling the reaction mixture, the solid is filtered off and washed with plenty of deionized water until the pH of the water layer is neutral. The collected solid is dried to obtain a mixture of 2,9-dibromopentacene 5,7,12,14-tetrone and 2,10-dibromopentacene 5,7,12,14-tetrone in 16% yield. IR: 1670 cm^{-1} strong ν(C=O) carbonyl, 1250 cm^{-1} broad δ(C-H) aromatic, 705 cm^{-1} strong γ(C-H) aromatic. ^{1}H-NMR (400 MHz, DMSO-d_6, 350 K): δ [ppm] = 8.94 (dd, *J* = 8.3, 5.9 Hz, 2H), 8.42 – 8.28 (m, 2H), 8.27 – 8.17 (m, 2H), 8.02 (m, 2H). ^{13}C-NMR (100 MHz, solid state, 298 K) δ [ppm] = 179.78, 136.09, 133.58, 128.15, 124.79.

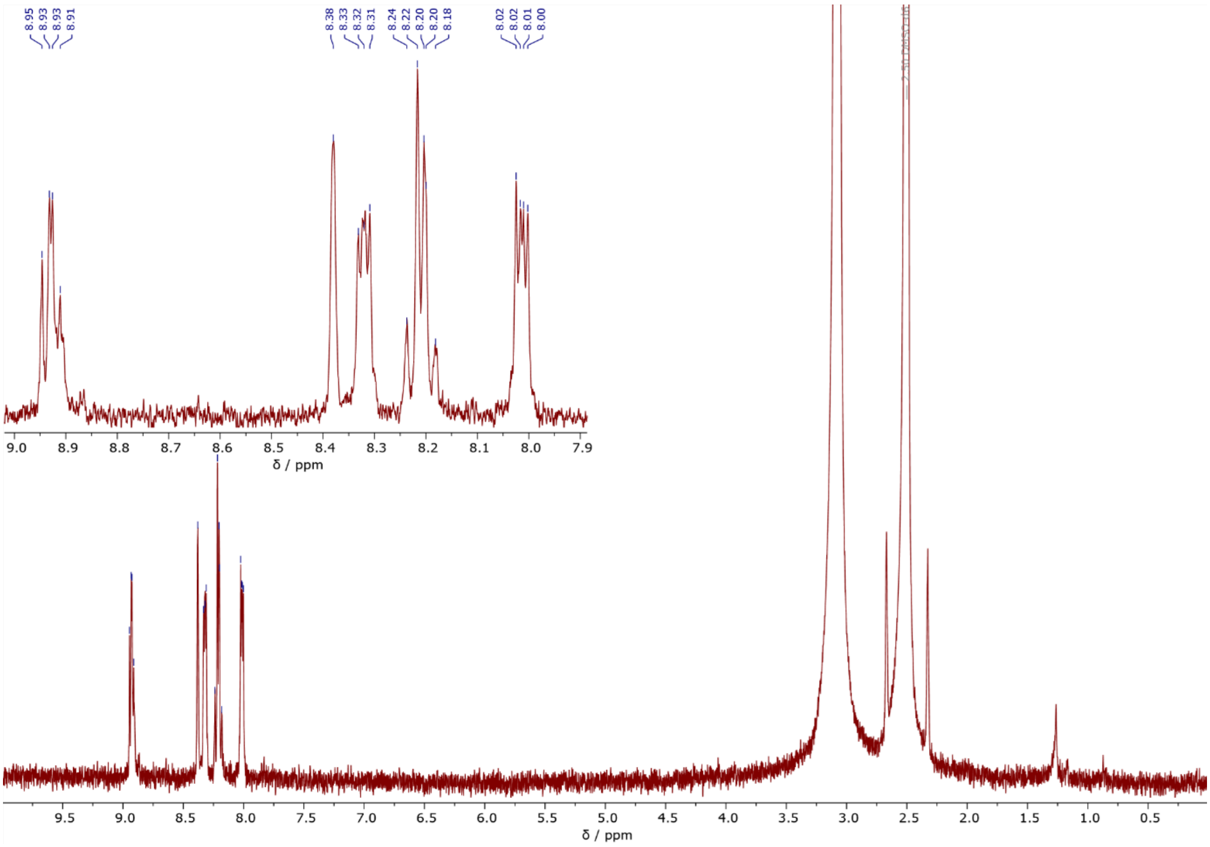

Figure S2 ^{1}H-NMR spectrum of the mixture 2,9-dibromopentacene-5,7,12,14-tetrone and 2,10-dibromopentacene-5,7,12,14-tetrone (400 MHz, DMSO-d_6, 350 K).

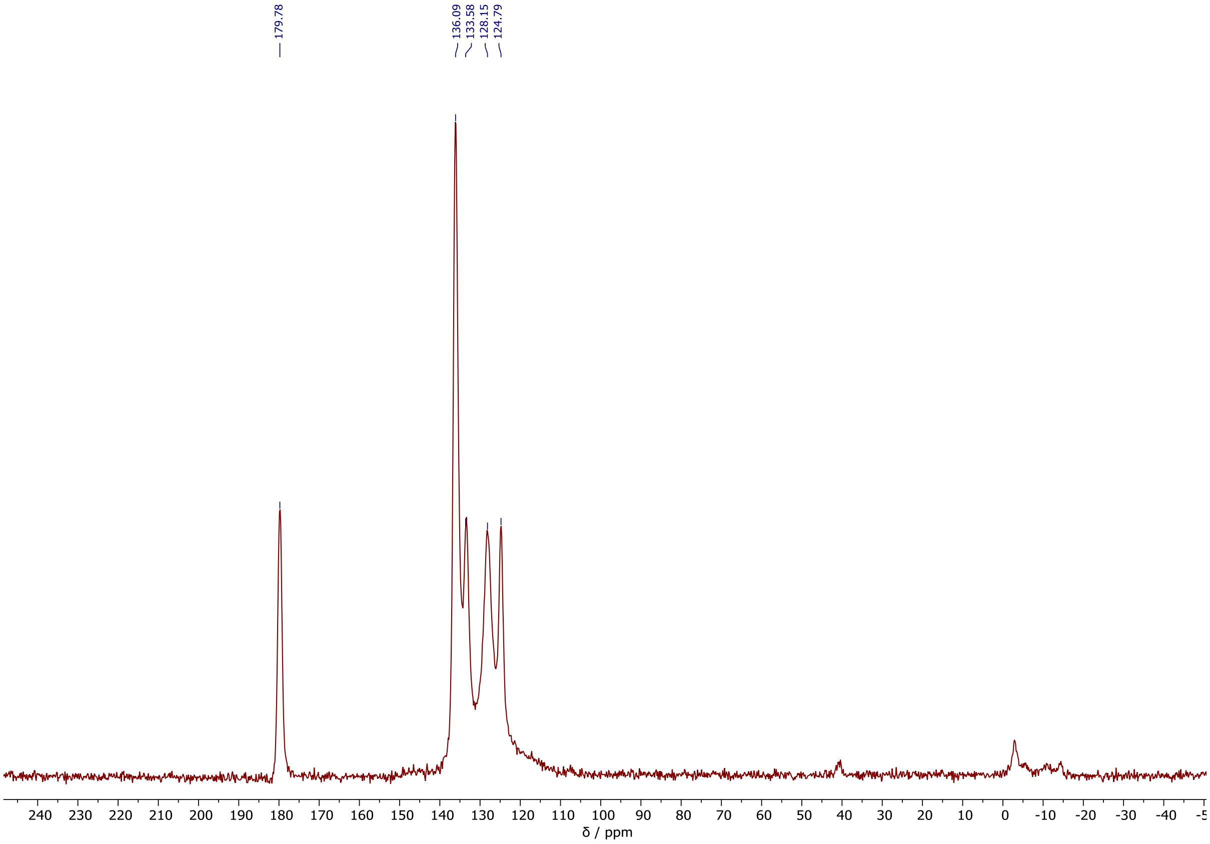

Figure S3 ^{13}C-NMR spectrum (solid state) of the isomeric monomer mixture (2,9-dibromopentacene5,7,12,14-tetrone and 2,10-dibromopentacene-5,7,12,14-tetrone).

4. Synthesis of poly(pentacenetetrone)

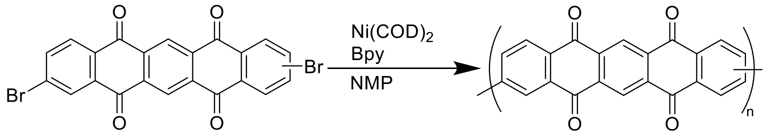

To a mixture of 2,9-dibromopentacene 5,7,12,14-tetrone and 2,10-dibromopentacene 5,7,12,14-tetrone (500 mg, 1 mmol) in a dry flask, were added 1,5-cyclooctadiene (250 µL, 0.002 mmol), 2,2′-bipyridin (320 mg, 2 mmol), and Ni(COD)$_2$ (250 mg, 0.91 mmol) under inert gas conditions. To this dry NMP solvent is added, and the flask is sealed under argon and heated to 80 °C for the first 4 hours and then to 200 °C for the next 12 hours. [2,3] After cooling the reaction mixture, the precipitated product is filtered off and washed with plenty of solvent and dried under vacuum to obtain a PPT in 47% yield. IR: 1670 cm^{-1} strong ν(C=O) carbonyl, 1250 cm^{-1} very broad δ(C-H) aromatic, 708 cm^{-1} γ(C-H) aromatic. ^{13}C-NMR (100 MHz, solid state, 298 K) δ [ppm] = 177.60, 133.12, 125.83. Elemental analysis: Calculated: C, 78.57; H, 2.49; N, 0; O, 19.03. Found: C, 68.69; H, 3.57; N, 3.05.

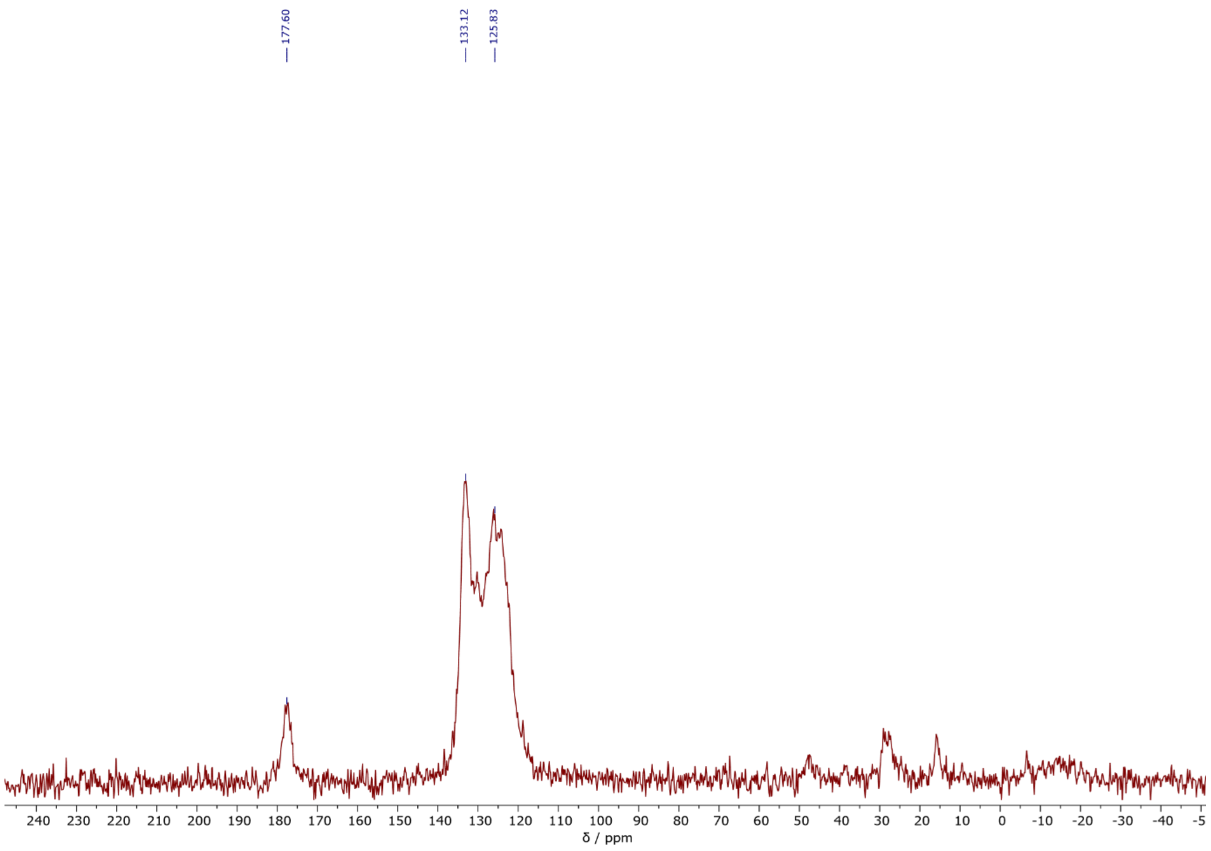

Figure S4 ^{13}C-NMR spectrum of poly(pentacenetetrone) (solid state).

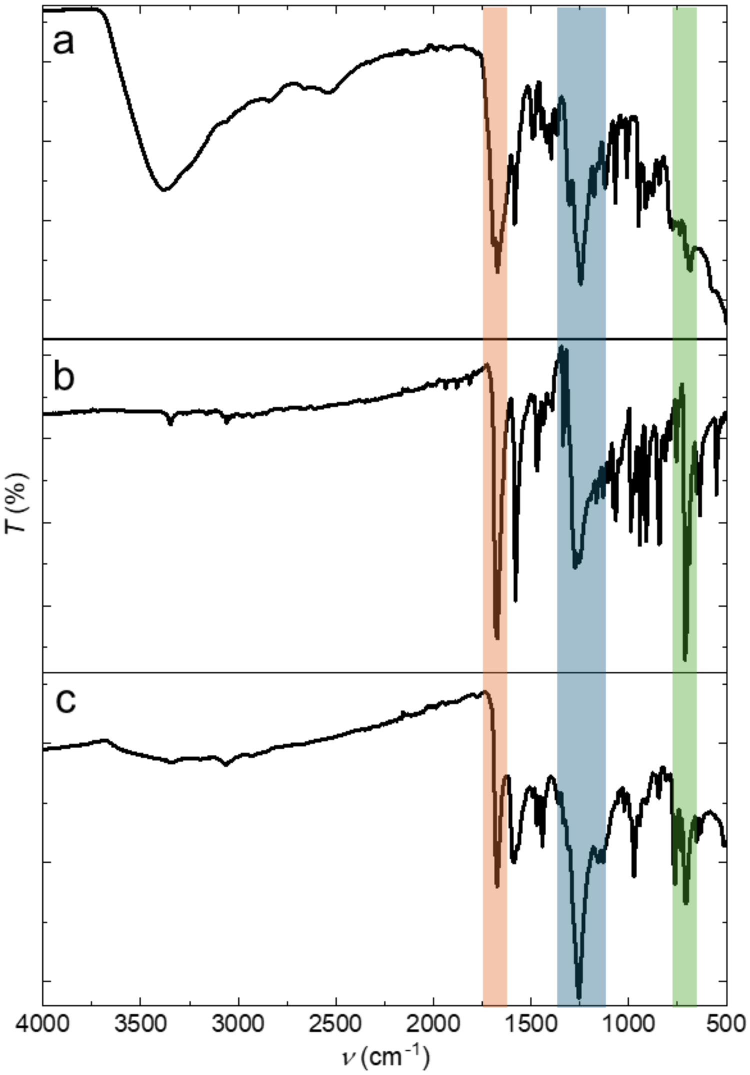

Figure S5 a. IR spectrum of the product of the first reaction step of the two isomers 2,5-bis(4-bromobenzoyl)terephthalic acid and 2,4-bis(4-bromobenzoyl)terephthalic acid, **b**. IR spectrum of the finished monomer $PTBr_2$ of the mixture of 2,9-dibromopentacene 5,7,12,14-tetrone and 2,10-dibromopentacene 5,7,12,14-tetrone, **c**. IR spectrum of the polymer PPT. The orange bar shows carbonyl-functionality and blue and green show the vibrations of the aromatics.

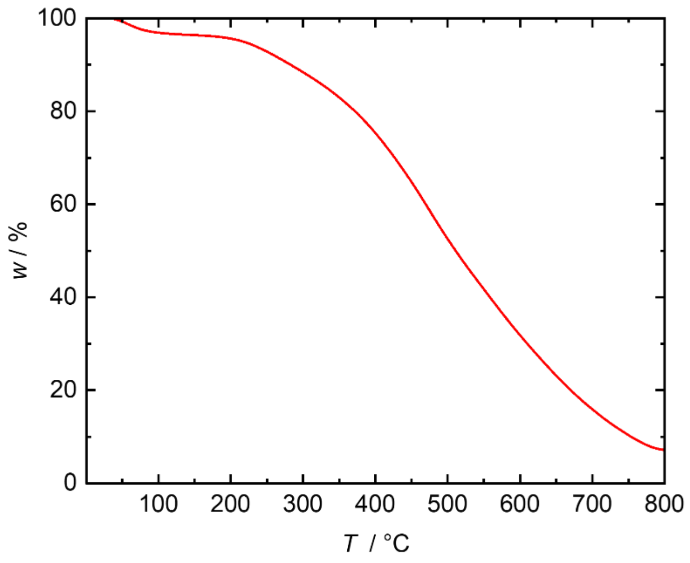

Figure S6 TGA measurement of PPT performed under N_2 atmosphere with a heating rate of 10 °C min^{-1}.

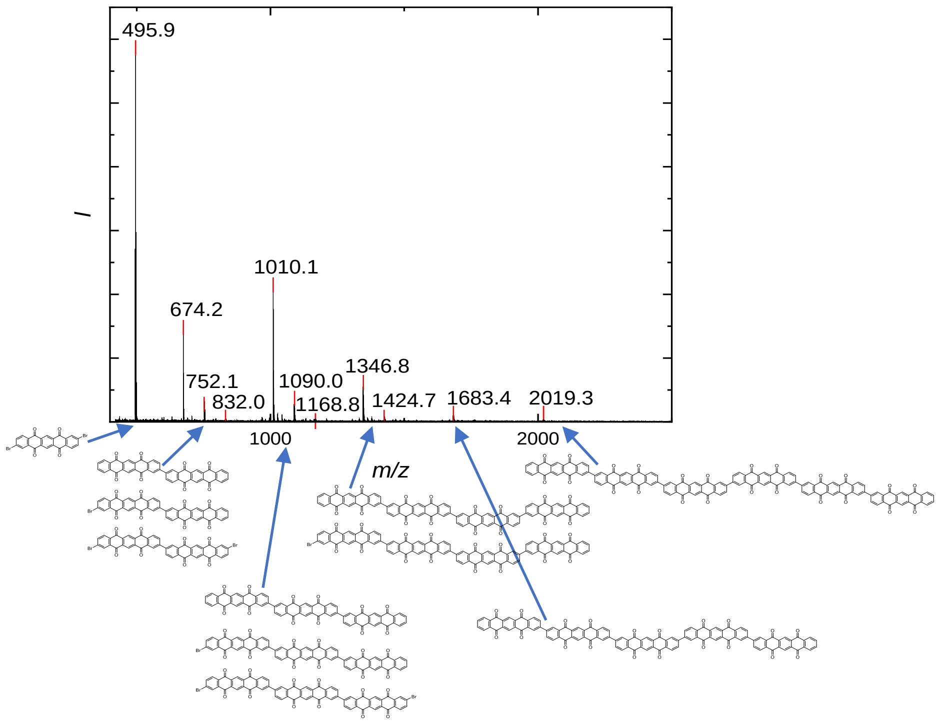

Figure S7 Mass spectrum of PPT in negative mode with assigned oligomers up to the hexamer.

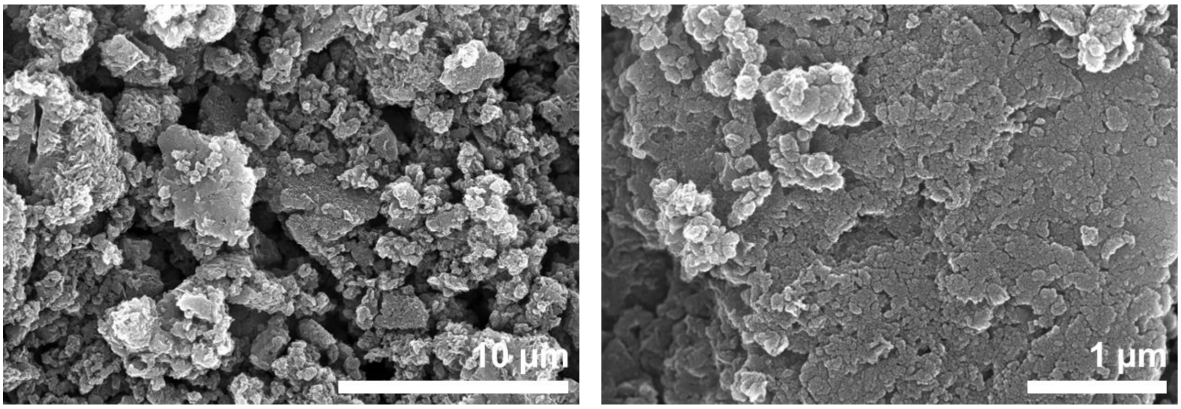

Figure S8 SEM images of the pure PPT polymer particles after synthesis.

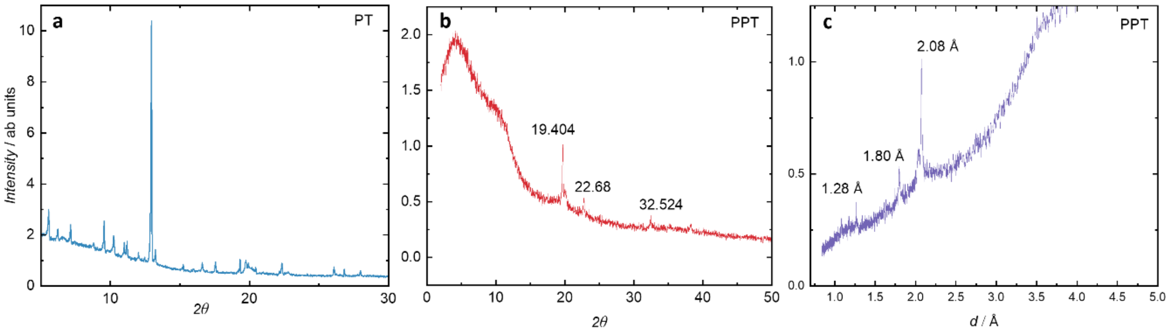

Figure S9 Powder XRD of **a.** PT with an intense and multiple small peaks indicating the crystalline nature of the PT monomer, **b** and **c.** PPT with an amorphous halo and few sharp peaks corresponding to intramolecular distances.

5. Synthesis of 2,9-dichloropentacene 5,7,12,14-tetrone and 2,10-dichloropentacene 5,7,12,14-tetrone.

The chlorinated monomer is prepared by following the same procedure used for the preparation of the brominated monomer, except that chlorobenzene was added in the first step instead of bromobenzene. IR: 1670 cm^{-1} strong ν(C=O) carbonyl, 1250 cm^{-1} broad δ(C-H) aromatic, 713 cm^{-1} strong γ(C-H) aromatic. ^{1}H-NMR (400 MHz, DMSO-d_6, 350 K): δ [ppm] = δ 8.95 – 8.85 (m, 2H), 8.35 – 8.24 (m, 3H), 8.08 – 7.90 (m, 3H).

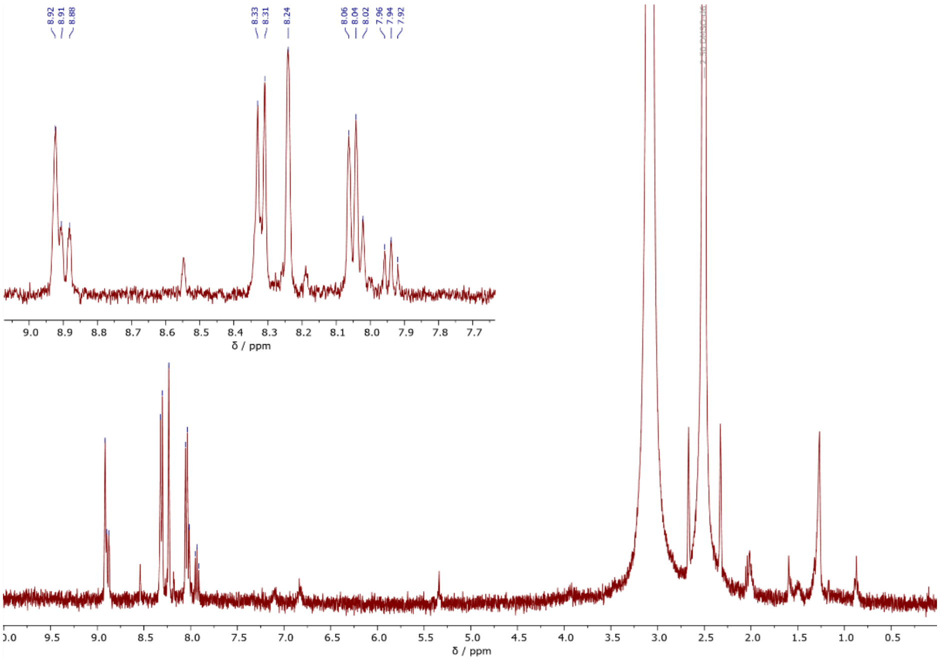

Figure S10 ^{1}H-NMR spectrum of the mixture 2,9-dichloropentacene 5,7,12,14-tetrone and 2,10-dichloropentacene 5,7,12,14-tetrone (400 MHz, DMSO-d_6, 350 K).

6. PPT free electrode preparation

The electrode is prepared following a similar procedure as for the PPT electrode preparation of A mixture of Super P and PVDF are added in 9: 1 ratio and ground thoroughly for 20 mins in a mortar and pestle to get a composite. The composite is transferred into a Thinky mixer capsule and a few drops of NMP were added. Then the capsule was subjected to centrifugation to get slurry of the composite. The slurry is cast on an Al foil using doctor blade technique and dried at 80 °C overnight to get uniformly coated composite. The coated foil is cut into circular electrodes of 12 mm diameter using a cutter knife. The cut electrodes are vacuum dried again at 120 °C for 3 h to remove traces of moisture accumulated on the electrode surface before using in coin cell fabrication. The dried electrode along with drying tube apparatus are transferred into glove box maintained at ≥ 0.1 ppm for O_2 and moisture.

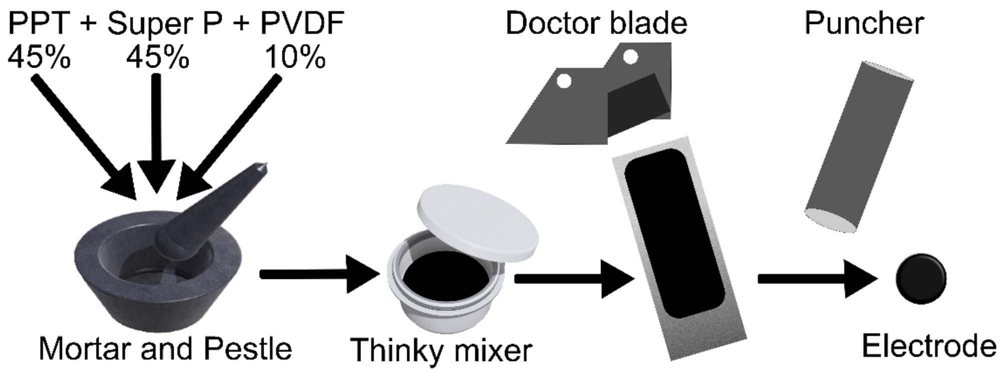

Figure S11 Scheme for the preparation of PPT electrode with a composition of PPT, Super P, and PVDF in the ratio 45:45:10

7. Choice of suitable electrolyte

Linear and cyclic carbonate solvents combined with lithium salts are widely studied as electrolytes for lithium (Li) batteries. Due to the significant similarities in properties between Li and sodium (Na) systems, carbonate-based electrolytes have also been investigated for sodium-ion batteries. Carbonate-based electrolytes, such as ethylene carbonate, propylene carbonate, ethyl methyl carbonate, dimethyl carbonate, and diethyl carbonate, form a passivating solid electrolyte interface (SEI) on Li battery anodes, preventing further electrolyte degradation. However, in sodium systems, the SEI dissolves due to the solubility of Na_2CO_3, unlike the stable Li_2CO_3 in lithium systems. This dissolution exposes more electrolyte to degradation during cell operation, as the reaction products fail to adhere to the electrode surface.

Glymes offer a promising alternative to carbonate-based electrolytes due to their stable electrochemical window. Compared to Li^+ ions, glymes exhibit a higher coordination number with Na^+ ions, providing stronger binding. This enhanced binding is attributed to the chelating effect of longer glymes, making tetraethylene glycol dimethyl ether (TEGDME) an ideal solvent for our study. The interaction between metal ions and the electrolyte significantly influences energy storage performance by forming carbon intercalating complexes, which improve charge-discharge efficiency.[4,5] Additionally, glymes are known to enhance rate kinetics compared to carbonate-based electrolytes.[6]

Studies report that 1 M solutions of $NaClO_4$, $NaPF_6$, and NaTFSI exhibit similar conductivities in propylene carbonate; however, $NaPF_6$ exhibits a superior electrochemical stability window.[4] Importantly, salts like NaFSI and NaTFSI can corrode aluminum (Al), often the

metal of choice as a substrate for the active materials. By contrast, $NaPF_6$ has no effect on Al, minimizing chemical interference with the cell components.[7] Considering these factors, 1 M $NaPF_6$ in TEGDME has been selected as the most suitable electrolyte for this study.

8. Electrochemical Characterizations

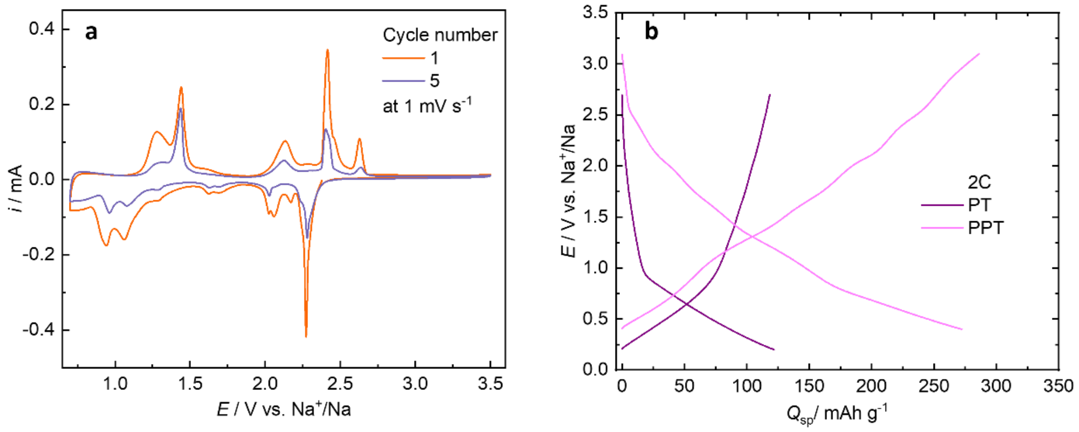

Figure S12 a. CV profile of PT cathode at 1 mV s^{-1} compared with their 1st and 5th cycle, **b.** charge-discharge profile comparison of PT and PPT cathodes at 2C.

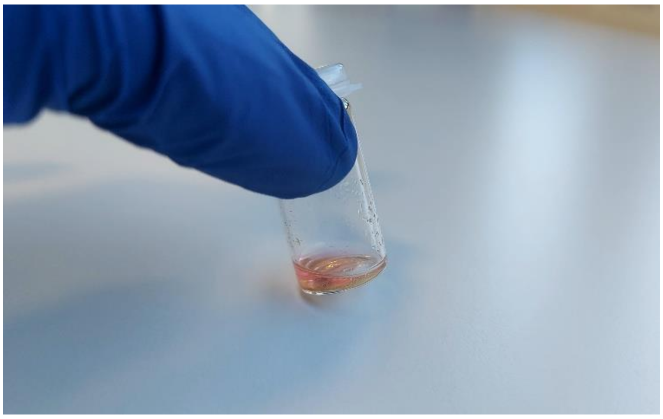

Figure S13 Dissolution of PT upon allowing the molecule to stand with 1 M $NaPF_6$ in TEGDME electrolyte over a period of 3 hours.

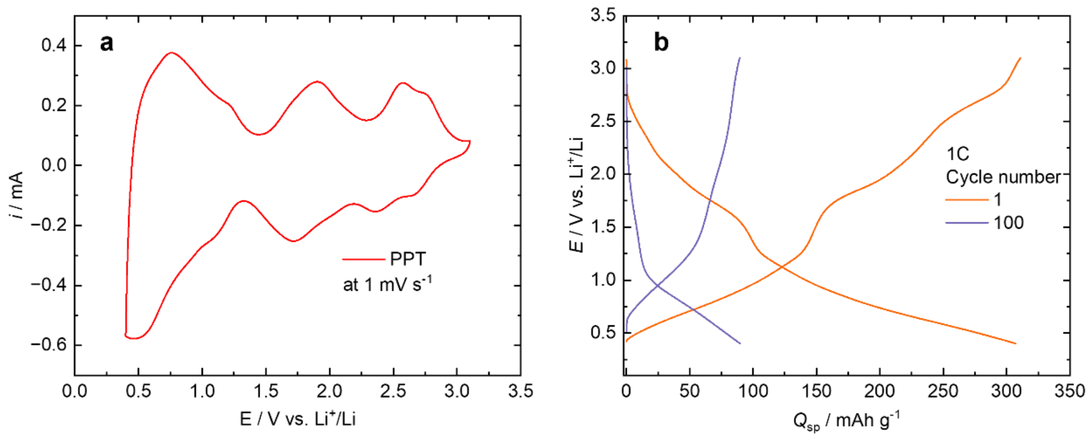

Figure S14 a. CV of the PPT cathode with Li anode at 1 mV s^{-1}, **b.** Charge-discharge curves of the same cell with 70% capacity fade observed between its 1st to 100th cycle at 1C.

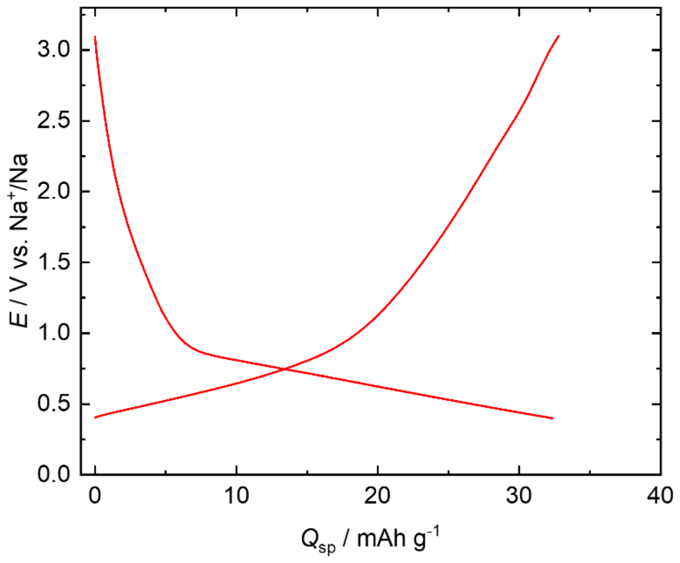

Figure S15 Galvanostatic charge-discharge profiles for estimating the charge contribution from Super P. The cell is composed of a Super P + PVDF composite cathode with a Na foil anode at 50 mA g^{-1}.

The capacity contribution of Super P to the cell is calculated by assuming there is 100% utilization of the active material (PPT) during cell operation. The contribution of the conductive additive (Super P) is calculated as follows:

Super P: 33 mAh for 1 g (from Figure S14)

PPT: 319 mAh for 1 g (calculated theoretical specific capacity)

(33x mAh) + (319x mAh) = Q_{sp} (experimentally determined total cell capacity)

x = Q_{sp} / 352

Contribution of capacity from Super P in any cell is 33x mAh g^{-1}.

For example, a cell with an experimental capacity Q_{sp} = of 314 mAh has a capacitive contribution of 29 mAh from Super P with active material and conductive carbon being in the same ratio.

Scheme for possible electrochemical reaction

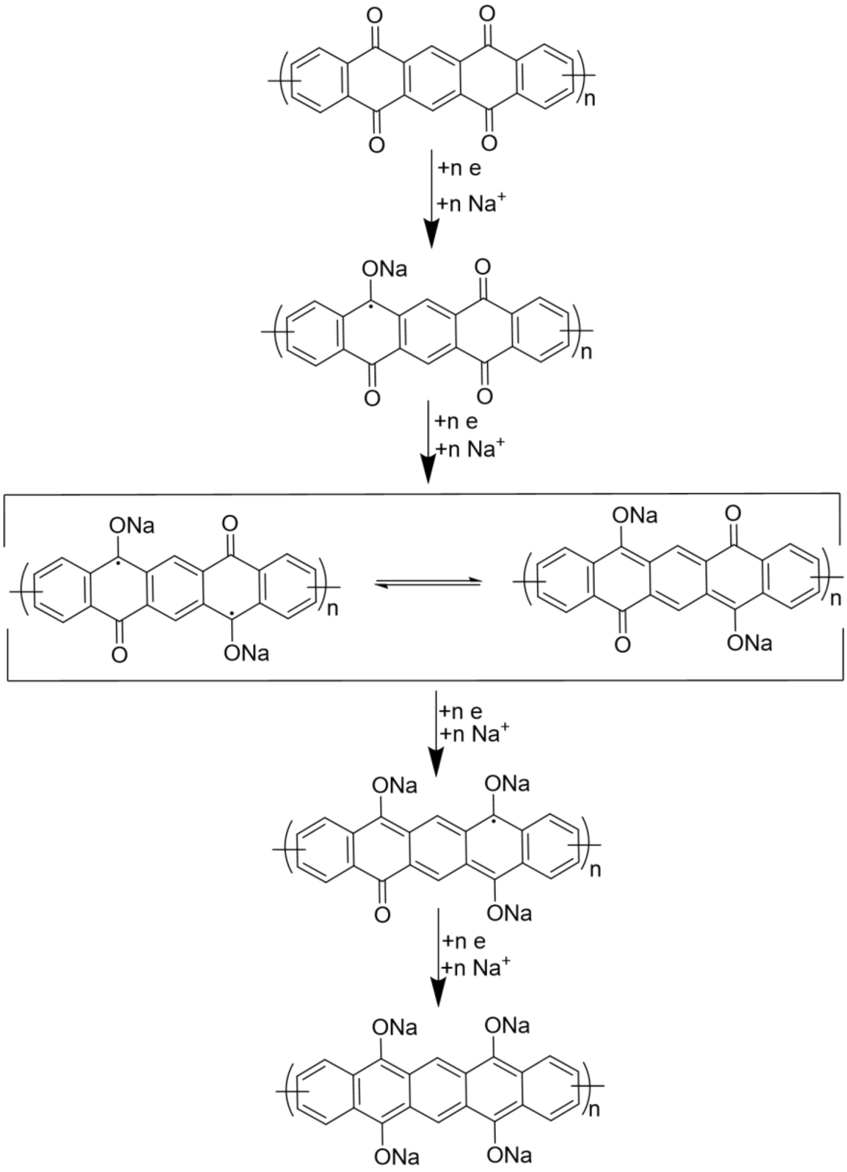

Scheme S1 Possible electrochemical process during the discharge process with 1 electron transfer involved in each redox step.

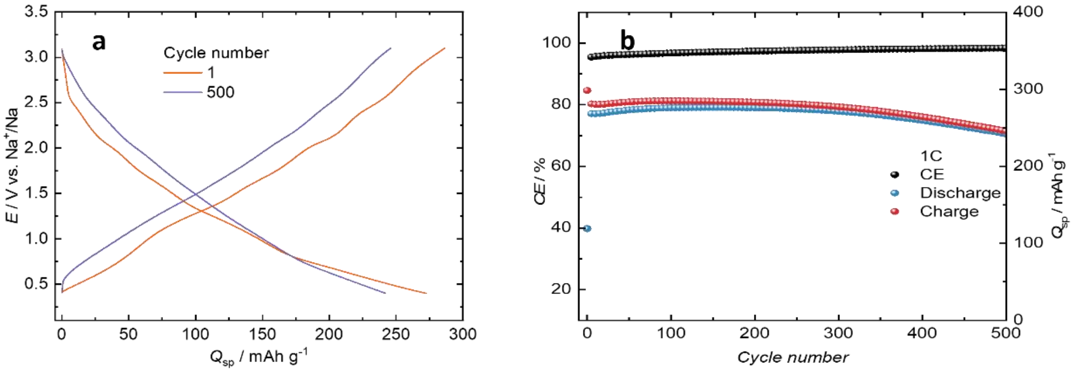

Figure S16 Charge-discharge of Na half-cell with PPT composite cathode at 1C with **a.** Capacity vs. cell voltage for 1st and 500th cycle, **b.** cycling performance of 500 cycles represented with Efficiency and Capacity vs. Cycle number.

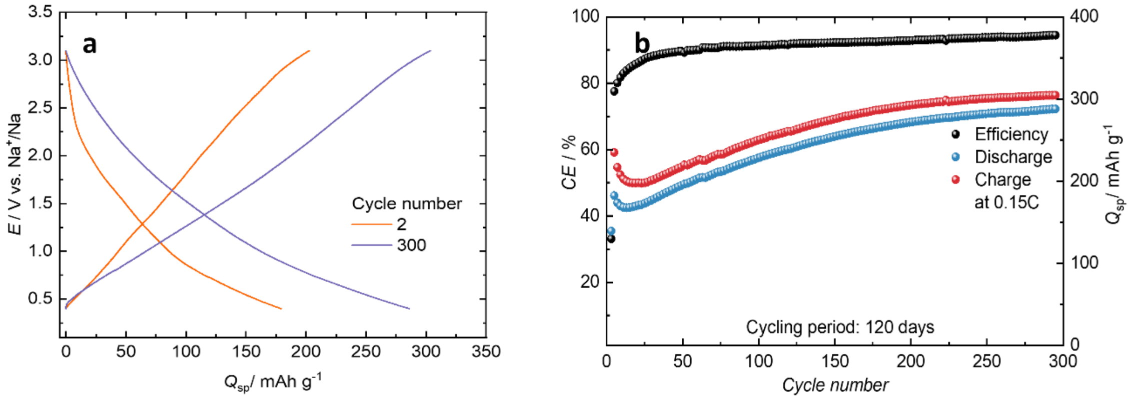

Figure S17 Galvanostatic charge-discharge of Na-PPT based cell at 0.15C **a.** with cell voltage vs. specific capacity, **b.** efficiency and specific capacity vs. cycle number up to 300, spanning a period of 120 days.

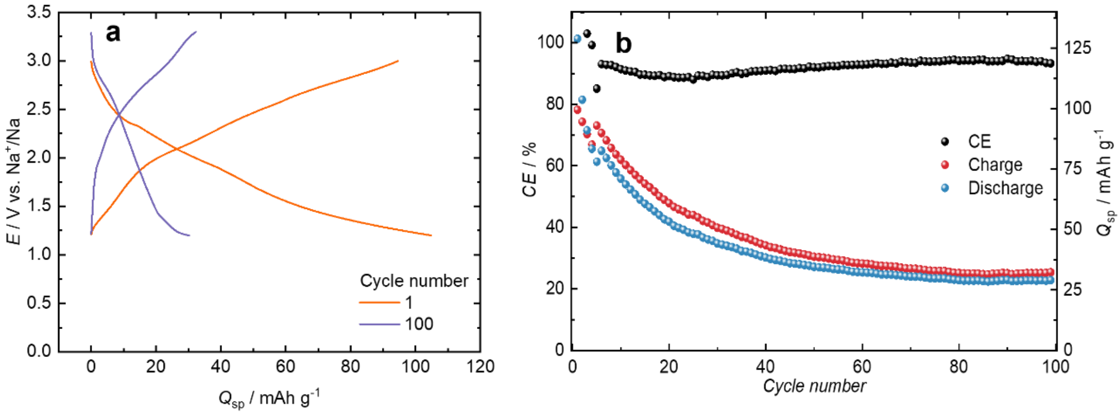

Figure S18 Galvanostatic charge-discharge at 1C for PPT synthesised from dichloro substituted monomer unit a Cell voltage vs. Specific capacity, b Coulombic efficiency and specific capacity vs. cycle number.

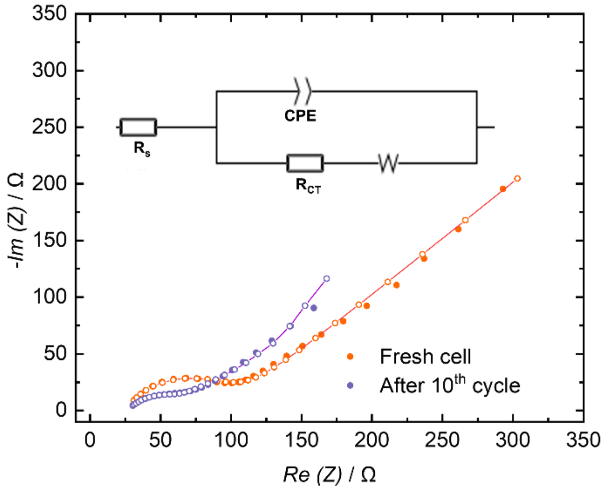

Figure S19 Nyquist plot for the PPT based cell recorded for the fresh cell and after 10 cycles of CV in the frequency range from 10 kHz to 100 mHz with closed circles indicating raw data and open circles with line indicating fitted data.

Table S1 Slopes of all the peaks in the log *i* vs. log *v* plots in Figure 3b

**Peaks**	***b* values**	
	**Oxidation (a)**	**Reduction (b)**
1	0.91	0.92
2	0.92	0.91
3	0.85	0.93
4	0.86	0.94

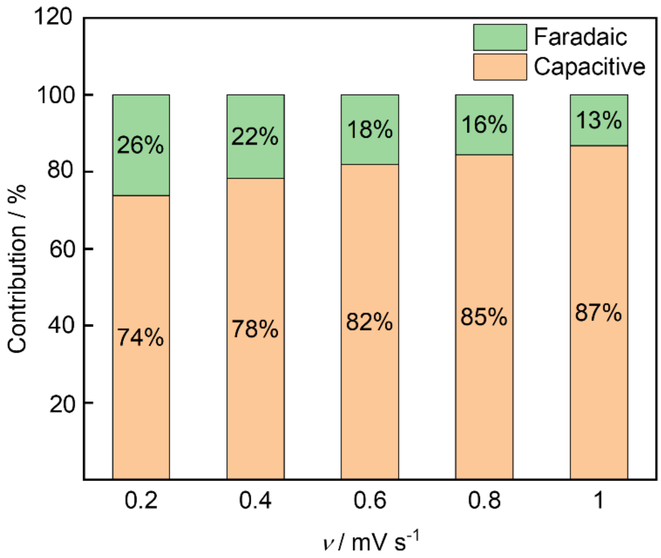

Figure S20 Bar diagram showing the faradaic and capacitive contribution for the PPT based cell at different *ν*.

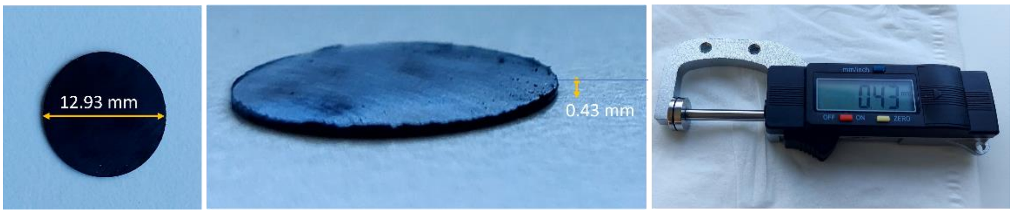

Figure S21 PPT pellet obtained by pressing for estimation of molar volume. Measured weight, diameter, and thickness of the pellet are 56.1 mg, 12.93 mm, and 0.43 mm respectively.

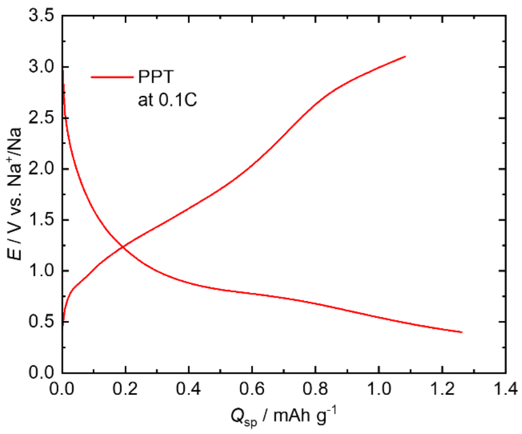

Figure S22 Charge-discharge of PPT based cell with cathode composition of 80:15:5 for PPT: Super P:PVDF at 0.1C.

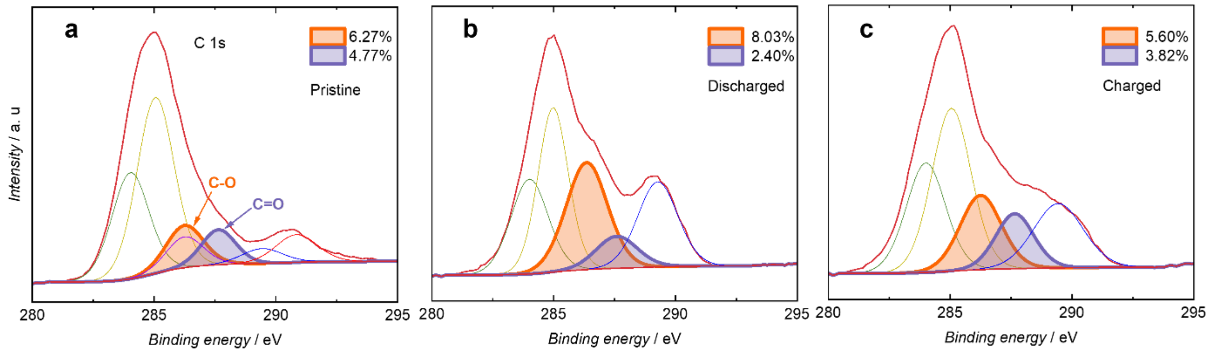

Figure S23 C 1s detailed XPS spectra of PPT-based composite cathodes in their **a.** pristine, **b.** discharged and **c.** charged state. The deconvolution of the envelope signal provides the individual species observed: green fit is C=C aromatic, yellow fit C-C/C-H aliphatic, orange fit C-O, purple fit C=O, blue fit O-C=O. For the pristine sample, two peaks with the same intensity were added, which account for the CH_2 (286.3 eV) fits violet and red for CF_2 groups of the PVDF binder.

Table S2 Comparison of the diffusion coefficients of Na^+ ions (D) for different organic and inorganic electrode materials.

**Material**	**Technique**	**Diffusion Coefficient *D* (cm² s⁻¹)**	**Ref.**
PPT	GITT	~1×10^{-10}	This work
PPTS	GITT	~1×10^{-9}	[7]
THQAP	GITT	1.5×10^{-11}	[8]
Na_2BNDI	GITT	1×10^{-11} - 7×10^{-11}	[9]
$Na_3V(PO_3)_3N$	GITT	1×10^{-13} - 1×10^{-11}	[10]
$NaFePO_4$	GITT	8.7×10^{-17}	[11]
	EIS	8.6×10^{-17}	[11]

Table S3 Comparison of organic materials with their electrochemical performance used in SBs

**Material**	**Composition of active material (additive, binder)**	**Low rate capacity, mAh g⁻¹ (C-rate)**	**High rate capacity, mAh g⁻¹ (C-rate),**	**Reversible capacity, cycle number (C-rate)**	**Ref.**
PPT	45% (Super P, PVDF)	314 (0.1)	70 (50)	255, 500 (2)	This work
PPTS	40% (Super P, PVDF)	290 (0.34)	155 (34)	230, 2000 (3.4)	[7]
2,6-PAQS	40% (Ketjen black, PTFE)	220 (0.2)	120 (50)	200, 1000 (4)	[12]
IEP-11-E12	50% (MWCNT, PVDF)	102 (1)	32 (60)	46, 9000 (2)	[13]

PBQS	60% (Ketjen black, PTFE)	282 (0.13)	200 (13)	155, 1000 (1.3)	[14]
C/$NaFePO_4$	82% (Carbon black, PVDF	120 (0.05)	23 (2)	90, 100 (0.1)	[15]
$Na_3V_2(PO_4)$ @C@rGO	80% (carbon nanotube, PVDF)	115 (1)	86 (100)	55, 10000 (100)	[16]

References

[1] E. Philippi, *Monatsh Chem* **1911**, *1*, 631.

[2] G. Machek, *Monatsh Chem* **1930**, *56*, 116.

[3] E. Philippi, F. Auslaender, *Monatsh Chem* **1921**, *42*, 1.

[4] B. Jache, J. O. Binder, T. Abe, P. Adelhelm, *Physical Chemistry Chemical Physics* **2016**, *18*, 14299.

[5] J. Maibach, F. Jeschull, D. Brandell, K. Edström, M. Valvo, *ACS Appl Mater Interfaces* **2017**, *9*, 12373.

[6] Y.-E. Zhu, L. Yang, X. Zhou, F. Li, J. Wei, Z. Zhou, *J Mater Chem A Mater* **2017**, *5*, 9528.

[7] M. Tang, S. Zhu, Z. Liu, C. Jiang, Y. Wu, H. Li, B. Wang, E. Wang, J. Ma, C. Wang, *Chem* **2018**, *4*, 2600.

[8] J. Zou, L. Ji, T. Xu, Q. Gou, S. Fang, P. Xue, M. Tang, C. Wang, Z. Wang, *J Colloid Interface Sci* **2024**, *676*, 715.

[9] Y. Wang, P. Bai, B. Li, C. Zhao, Z. Chen, M. Li, H. Su, J. Yang, Y. Xu, *Adv Energy Mater* **2021**, *11*, 2101972.

[10] M. Chen, W. Hua, J. Xiao, D. Cortie, X. Guo, E. Wang, Q. Gu, Z. Hu, S. Indris, X. Wang, *Angewandte Chemie* **2020**, *132*, 2470.

[11] Y. Zhu, Y. Xu, Y. Liu, C. Luo, C. Wang, *Nanoscale* **2013**, *5*, 780.

[12] B. Flamme, B. Jismy, M. Abarbri, M. Anouti, *Mater Adv* **2021**, *2*, 376.

[13] A. Molina, N. Patil, E. Ventosa, M. Liras, J. Palma, R. Marcilla, *Adv Funct Mater* **2020**, *30*, 1908074.

[14] Z. Song, Y. Qian, T. Zhang, M. Otani, H. Zhou, *Advanced Science* **2015**, *2*, 1500124.

[15] Y. Zhu, Y. Xu, Y. Liu, C. Luo, C. Wang, *Nanoscale* **2013**, *5*, 780.

[16] X. Rui, W. Sun, C. Wu, Y. Yu, Q. Yan, *Advanced Materials* **2015**, *27*, 6670.
